# Psychological and Educational Intervention to Improve Tuberculosis Treatment Adherence in Ethiopia Based on Health Belief Model: A Cluster Randomized Control Trial

**DOI:** 10.1371/journal.pone.0155147

**Published:** 2016-05-11

**Authors:** Habteyes Hailu Tola, Davoud Shojaeizadeh, Azar Tol, Gholamreza Garmaroudi, Mir Saeed Yekaninejad, Abebaw Kebede, Luche Tadesse Ejeta, Desta Kassa, Eveline Klinkenberg

**Affiliations:** 1 Tehran University of Medical Sciences-International Campus, School of Public Health, Department of Health Education and Promotion, Tehran, Iran; 2 Ethiopian Public Health Institute, TB/HIV Research Directorate, Addis Ababa, Ethiopia; 3 Tehran University of Medical Sciences International Campus, School of Public Health, Department of Epidemiology and Biostatistics, Tehran, Iran; 4 KNCV Tuberculosis Foundation, The Hague, The Netherlands; 5 Department of Global Health, Academic Medical Centre, University of Amsterdam, Amsterdam Institute for Global Health and Development, Amsterdam, The Netherlands; University College London, UNITED KINGDOM

## Abstract

**Background:**

Treatment non-adherence results in treatment failure, prolonged transmission of disease and emergence of drug resistance. Although the problem widely investigated, there remains an information gap on the effectiveness of different methods to improve treatment adherence and the predictors of non-adherence in resource limited countries based on theoretical models. This study aimed to evaluate the impact of psychological counseling and educational intervention on tuberculosis (TB) treatment adherence based on Health Belief Model (HBM).

**Methodology:**

A cluster randomized control trial was conducted in Addis Ababa from May to December, 2014. Patients were enrolled into study consecutively from 30 randomly selected Health Centers (HCs) (14 HCs intervention and 16 HCs control groups). A total of 698 TB patients, who were on treatment for one month to two months were enrolled. A structured questionnaire was administered to both groups of patients at baseline and endpoint of study. Control participants received routine directly-observed anti-TB therapy and the intervention group additionally received combined psychological counseling and adherence education. Treatment non-adherence level was the main outcome of the study, and multilevel logistic regression was employed to assess the impact of intervention on treatment adherence.

**Results:**

At enrollment, the level of non-adherence among intervention (19.4%) and control (19.6%) groups was almost the same. However, after intervention, non-adherence level decreased among intervention group from 19.4 (at baseline) to 9.5% (at endpoint), while it increased among control group from 19.4% (baseline) to 25.4% (endpoint). Psychological counseling and educational interventions resulted in significant difference with regard to non-adherence level between intervention and control groups (Adjusted OR = 0.31, 95% Confidence Interval (CI) (0.18–0.53), *p < 0*.*001*)).

**Conclusion:**

Psychological counseling and educational interventions, which were guided by HBM, significantly decreased treatment non-adherence level among intervention group. Provision of psychological counseling and health education to TB patients who are on regular treatment is recommended. This could be best achieved if these interventions are guided by behavioral theories and incorporated into the routine TB treatment strategy.

**Trial Registration:**

Pan African Clinical Trials Registry PACTR201506001175423

## Introduction

Tuberculosis (TB) treatment non-adherence is one of challenging conditions, because its risk of prolonged disease transmission, treatment failure and occurrence of drug resistance. Evidences suggests that despite complete treatment interruption (default), intermittent interruption is also a point of concern, since non-adherence of treatment can leads to poor treatment outcomes [1,2]. Medication non-adherence has several socioeconomic and health consequences [3–7]. Treatment non-adherence causes among other factors, hospital beds to be occupied for too long by many patients [3,4,6], economic depletion [4,5], prolonged morbidity [6], induces psychological disorder [3,5] and increases risk of mortality [3,7]. Apart from immuno-pathological and pharmacological impacts, treatment intermittency or non-adherence remains an important cause of Multidrug Resistant and Extensively Drug Resistant TB (MDR-TB and XDR-TB) bacilli strain development [2]. For instance, based on a previous study, the chance of developing MDR-TB among those who interrupted the treatment (for at least for one day) is 13 times that of individuals who did not interrupt the treatment at all [2]. Because of treatment non-adherence and biopharmacological effect of TB treatment on MDR-TB, the global prevalence of MDR-TB is increasing. According to World Health Organization’s (WHO) recent report, globally, MDR-TB prevalence among new cases of TB is 3.6%, while among previously treated TB cases is 20.2% [8].

Studies show that a number of factors are influencing TB-treatment adherence. Among these factors, knowledge about TB and its treatment, distance to nearest health facility, perceived stigma, perception about disease and its treatment, psychological distress, change of residential place, and economic status are some [9–22]. In order to decrease non-adherence level, various interventions designed and implemented across the world [23–25]. For instance, an intervention that carried out based on enhanced TB adherence model, which focused on patient and professional empowerment, proved to positively impact TB treatment adherence [23]. Furthermore, a study conducted in east Kazakhstan shown the effectiveness of psychosocial support for MDR-TB patients in improving treatment adherence [24]. On the other hand, educational intervention that was provided by physicians at low socioeconomic setting in Bangladesh increased the cumulative adherence among intervention compared to control groups [25]. However, these interventions are limited to particular sociocultural settings hence need to replicate them in different areas.

Although evidence suggests that public health and health promotion interventions that are based on behavioral science theories are more effective than those without theoretical model [26,27], available interventional studies related to TB treatment adherence lack theoretical model as guides. Moreover, there are no considerable interventions that have been implemented to assess the applicability of theoretical models for TB treatment adherence promotion through targeting factors influencing patients’ adherence behavior in some sociocultural contexts. Ethiopia is one of those countries where such information is lacking despite TB treatment non-adherence is being high (ranging from 10% to 21%) [9–11], and the prevalence of MDR-TB is also increasing from 1.6% to 2.3% among new cases and from 12% to 17.8% among previously treated cases [28,29]. Thus, evaluating interventional experiences based on theoretical model in particular sociocultural context to promote TB treatment adherence is essential. Therefore, this study was aimed to evaluate a combined psychological counseling and educational intervention in order to decrease TB treatment non-adherence based on HBM in Ethiopian sociocultural context.

HBM is recommended as useful model to comprehend and explain health behaviors including treatment adherence that need to be practiced by patients [30,31] and it is also useful to plan behavioral interventions including treatment adherence [27]. However, HBM is being criticized for putting emphasis on individual characteristics and cognitive factors, giving less attention to social influences and emotional components of behavior [32]. HBM is a theoretical model that has been constructed from six domains [32]. These domains are perceived susceptibility, perceived severity, perceived barriers, perceived benefit, cue to action and perceived self-efficacy. According to the concept of HBM, patients on TB treatment are likely to adhere to their medicinal regimen under a specific five sets of conditions [31,33]. First, patients must have some minimal level of TB disease knowledge and motivation towards staying free of TB. Second, patients must perceive themselves as vulnerable to TB and they must also be convinced that TB is a serious medical and health problem. Third, patients must also be convinced that being on TB treatment and adherence are effective to cure TB, that it is indeed possible to obtain control over the problems at acceptable psychological or tangible social barriers and that the barriers do not outweigh the benefits of TB treatment. Fourth, the presence of an internal or external stimulus, referred as “cue to action,” that triggers health behavior of patients such as taking TB medication. Finally, patients’ self-efficacy belief to strictly follow TB treatment should be maintained till the final treatment period.

## Materials and Methods

### Study Setting

This study was conducted among all forms of TB patients with or without HIV diagnosed or referred to 30 selected Health Centers (HCs) in Addis Ababa, a capital city of Ethiopia. Addis Ababa has an estimated total population of 2,975,608 [34]. Each HC in Ethiopia is supposed to serve 25,000 people who live within catchment area assigned that HC [35]. All diagnosed TB patients are treated under directly observed treatment short course (DOTS) strategy, according to national TB-HIV and leprosy treatment guideline [36], and the treatment is free of charge.

### Study Design, Sampling and Data Collection

**Fig 1:**
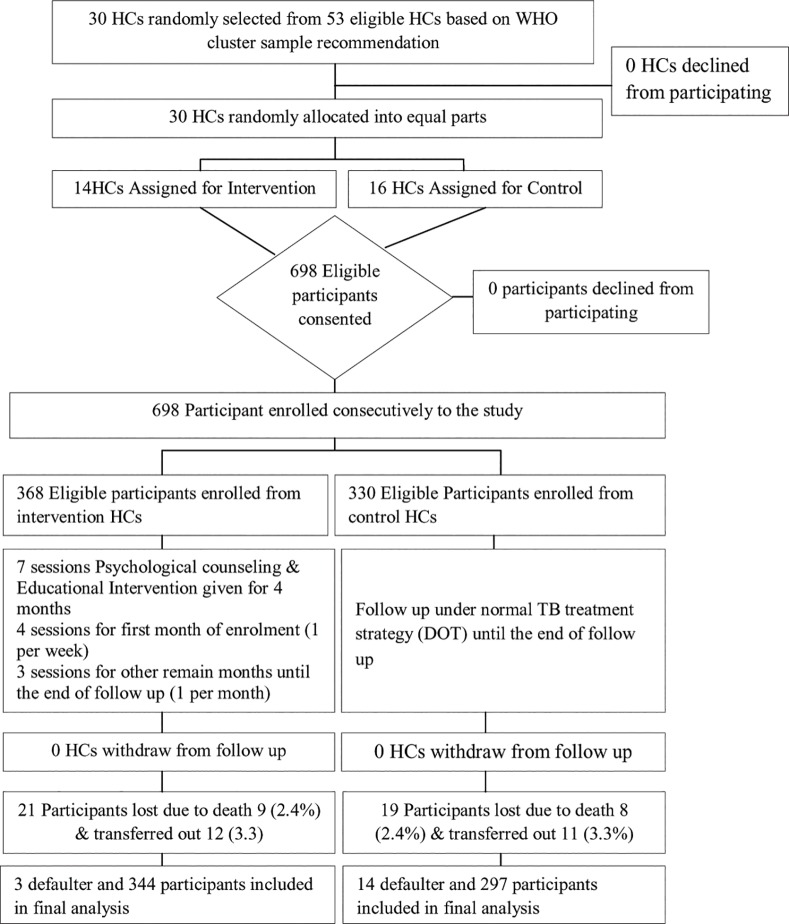
Cluster Randomized Control Trial Flow Diagram.

A cluster Randomized Control Trial (RCT) study was conducted among all TB patients who were on first line and MDR-TB treatment regimens under DOTS strategy in Addis Ababa, from May to December, 2014. Addis Ababa has 10 sub-cities with a total of 53 HCs and 10 public hospitals. As shown in Fig 1, a cluster of 30 HCs were selected by simple random sampling technique from 53 HCs which provide TB treatment under normal DOTS strategy based on WHO cluster sampling recommendation [37]. Thirty HCs were divided into two groups using block randomization method and 4 block sizes were used. Since each HC serves only a population within their catchment area, participants who selected in control and intervention groups are far from each other, this could avoid information contamination.

The major inclusion criteria of the study were: TB patients who were on treatment for one month -to-two months, above 17 years old, not participating in similar study, mentally capable to provide consent, and physically capable to follow intervention. Patients fulfilling the criteria were enrolled consecutively from each HC based on quota sampling method at selected HCs. TB patients who had been on treatment for 1–2 months were enrolled in this study, and this was made so as to measure a baseline psychological distress as recommended by Kessler-10 (K-10) items instrument to measure psychological distress within the past one month [38]. In addition, since there is a disagreement between different authors on the actual duration of symptoms necessary to make a diagnosis of generalized anxiety disorder, (i.e. one month to six months) [39,40] we took into account one month of duration (from date of diagnosis for TB) to measure psychological distress. Similarly, Visual Analogue Scale (VAS) measures treatment non-adherence level of patient’s in the past one month [41]. Thus, due to these reasons, we were obliged to enroll study participants one month after commencement of their treatment, even though beginning intervention from the first date of diagnosis and initiation of treatment is recommended to decrease non-adherence level among TB patients on treatment.

Structured questionnaires were designed and used to assess patients TB knowledge and the perception scores were based on the six HBM domains. To assess the presence of psychological distress within the last one month K-10 items scale was used. In addition, WHO Alcohol Use Disorder Identification Test (AUDIT) was used to assess Alcohol Use Disorder (AUD) risk. Both designed and standard questionnaires were validated before actual data collection by test and retest pilot study at selected study sites and they were valid at the recommended (70%) Cronbach’s alpha (α).

Classical VAS was used to assess patients’ non-adherence level. Although, VAS is not the gold standard tool to measure treatment non-adherence, it is recommended as a useful tool for screening patient’s non-adherence level in resource limited setting and it is relatively less influenced by response bias [41]. Non-adherence level of patients was estimated by participants themselves on VAS after through explanation and was given to patients at enrolment and end of follow up. Participants guessed their non-adherence level on VAS, labeling from 0% (not single dose or schedule missed) to 100% (not single dose taken) after they were asked the question “how many of your schedule or medication did you miss in the last 30 days (from percent)?” Participants who estimated their non-adherence level at 10% and above were considered as non-adherent based on WHO non-adherence definition [42] and national TB-HIV and leprosy treatment guideline [36]. In addition, patients who had been on treatment for at least one month and who interrupted the treatment for two months and more consecutively were considered as defaulters [36]. Both defaulters and interrupters (patients who missed a dose) were encouraged to continue their treatment upon their return to treatment center. The treatment protocol for defaulters and interrupters is either providing them with the missed dose or re-initiating the treatment as beginners based on the sputum result (as recommended in national treatment guideline), and then making them to complete the treatment [36]. Thus, in this study, non-adherent patients meant: (1) those who interrupted their treatment and estimated their non-adherence level greater than or equal to 10% on VAS, (2) those who interrupted their treatment for more than 10 doses due to medication adverse effects, and (3) those who defaulted from the treatment. Non-adherence level data collected (in percentage) was re-coded into a dichotomous variable: adherent and non-adherent for analysis.

Intervention that was intended to reduce non-adherence level, targeting TB patients on treatment, had two components: (1) anxiety and depression counseling (psychological distress counseling); and (2) patient education based on six HBM domains concept. TB patients were counseled for anxiety and depression management method to help them follow their treatment firmly during the entire period of treatment (from second month to sixth month of treatment initiation) [Fig 1]. In addition, a period of four months was dedicated (beginning from second month of treatment initiation) to health education on TB disease acquisition, the risks and consequences associated with non-adherence behavior, methods to help overcome psychological barriers to follow up the treatment, benefits of TB medication and treatment adherence, and methods to develop self-efficacy. These health education interventions were provided by trained health professionals at each TB clinic; once in a week for the first month of enrollment, and once in a month until the end of TB treatment, in total seven sessions [Fig 1]. Basing from the Bangladesh experience, time regimen of intervention provision was set at 30 minutes [31]. Patients recruited into the control group were made to receive usual DOTS services, and there were no anxiety and depression counseling and education interventions in the entire period of the study [Fig 1]. Intervention ended after four months of beginning the intervention because full dose of TB treatment ends at sixth month of treatment initiation for new cases. The primary outcome of the study was TB treatment non-adherence, and the secondary outcomes were psychological distress, knowledge and change of six HBM domains. Both the primary and secondary outcomes were measured at baseline (first to second month of treatment initiation) and at endpoint (sixth month of treatment initiation).

Ethical approval was obtained from Tehran University of Medical Sciences-International Campus, Ethiopian Public Health Institute and Addis Ababa City Administration Health Bureau Ethical Review Boards. All study participants enrolled to this study were 18 years or older, and mentally capable to provide written informed consent independently. Both oral and written informed consent was obtained from each study participant at recruitment. This trial was registered in Pan African WHO trial registry (www.pactr.org) retrospectively and its unique identification number is PACTR201506001175423.

### Data Analysis

The effect of intervention was measured at endpoint of follow up, using endpoint non-adherence level, while adjusting for baseline characteristics and clusters. Data cleaning and descriptive analyses were performed using IBM SPSS software version 20, while multilevel logistic regression analysis was conducted with MLwin 2.10 software. Baseline participants’ characteristics including non-adherence level were compared between intervention and control groups. Two-level multilevel logistic regression analysis was conducted to compare baseline HBM domains and knowledge level between two groups (control and intervention) while adjusting for clusters, psychological distress and other sociodemographic variables. In two-level multilevel logistic regression model, patients were in the first level and HCs were in the second level. All independent variables had p value less than 0.20 on unadjusted model, and all baseline HBM domains were included in the final multilevel logistic regression model. For additional model clarification, five HBM domains with p value greater than 0.2 (on unadjusted model) were removed from the model alongside with other variables with p value greater than 0.2. In all models, Wald tests were used to test the hypotheses, and to calculate the p values. In forward variable selection multilevel logistic regression, Penalized Quasi Likelihood (PQL) approximation was used for model estimation, and evaluating the clustering effect of centers in two-level model and the effect of all HBM domains simultaneously, while adjusting for clusters. All analyses were conducted based on intention to treat which is appropriate for a cluster randomized design study data analysis. [43]. Though missing proportion was not different between intervention and control groups [Fig 1], complete case analysis with regression adjustment for baseline covariate technique was used to address missing data related problems. Moreover, since participants those who missed were not different from participants that remained regarding sociodemographic variables [Table 1], they less likely influence the results.

**Table 1:** Baseline Participants’ Characteristics.

Characteristics		Study Groups			
		Intervention [N = 368]		Control [N = 330]	
		Frequency	% (95% CI)	Frequency	% (95% CI)
	Female	158	43 (38–48)	139	42(37–48)
Gender	Male	210	57(52–62)	191	58(53–63)
	18–24	98	27(22–31)	95	29(24–34)
	25–34	144	39(34–44)	120	36(31–42)
Age Group (in year)	35–44	72	20(16–24)	64	19(16–24)
	≥45	54	15(11–19)	50	15(12–19)
Education Status	Elementary & Less	213	58(53–63)	177	54(48–60)
	High School	119	32(28–37)	105	32(27–37)
	Diploma & Above	36	10(7–13)	48	15(11–19)
	Married	123	33(29–38)	101	31(26–36)
Marital Status	Unmarried	245	67(62–71)	229	69(64–74)
	Employed	175	48(43–53)	150	46(40–51)
Employment Status	Unemployed	141	38(34–43)	153	46(41–52)
	Daily Labor	52	14(11–18)	27	8(6–12)
	None Reactive	274	75(70–79)	274	83(79–87)
HIV Serostatus	Reactive	94	26(21–30)	56	17 (13–21)
ART Status	Not on ART	286	78(73–82)	231	70(65–75)
	On ART	61	18(13–21)	42	13(10–17)
	New	279	76(71–80)	248	75(70–80)
TB Treatment History	Previously Treated	89	24(20–29)	82	25(21–30)
	Pulmonary	228	62(60–67)	188	57(52–62)
TB Type	Extra Pulmonary	126	34(30–39)	89	27(23–32)
	MDR-TB	14	4(2–6)	53	16(13–20)
Current Smoking	None Smoker	335	91(88–94)	303	92(88–94)
History	Smoker	33	9(6–12)	27	8(6–12)
Distance to HC (in	Within Catchment	352	96(93–97)	302	92(88–94)
km)	Out of Catchment	16	4(3–7)	28	9(6–12)
Alcohol Use Disorder	No Risk	307	83(79–87)	286	87(83–80)
	There is Risk	61	17(13–21)	44	13(10–18)
Psychological	No Symptom	204	55(50–60)	153	46(41–52)
Distress Symptom	There is Symptom	164	45(40–50)	177	54(48–59)
	Low(0–4)	138	40(33–43)	137	42(36–47)
Economic Status	Medium (5–6)	103	30(24–33)	66	20(16–25)
	High(7–10)	106	31(24–34)	70	21(17–26)

ART- Antiretroviral Therapy

HIV- Human Immunodeficiency Virus

Non-adherence proportion under normal DOTS treatment that was reported previously from study area was 20% [9] and this data was used as true proportion to determine sample size. In addition, estimated precision of 5%, 1.5 design effect, 0.05 Inter-class Correlation Coefficient (ICC) based on pilot study result, 10 cluster size, 80% power and 10% contingency sample were considered for total sample size estimation. Consequently, 684 (342 for each group) participants were determined to be included into the study. However, 368 participants were enrolled into intervention group and 330 participants into control group [Fig 1] so as to include patients who fulfilled our inclusion criteria at selected HCs.

## Results

### Participants’ Characteristics

A total of 368 participants were enrolled into intervention group and they were provided with psychological counseling and educational intervention, the total sessions being seven, till the end of treatment. Whereas 330 participants were enrolled into control group and provided with no intervention except follow up under normal Directly Observed Therapy (DOT) treatment schedule [Table 1, Fig 1]. The total follow up period was four months and the follow-up started from second month of treatment to sixth month of treatment initiation. The follow up ended at sixth month of TB treatment initiation, since the total duration of treatment for newly diagnosed TB patient is six months under DOTS strategy. However, non-adherent and MDR-TB patients were not followed-up till the end of their treatment period; rather it was their non-adherence level that was measured till the end of the study follow-up period.

At the end of follow up, 658 (94%) total participants (344 from intervention group, 297 from control group and 17 defaulters) were included in the analysis. However, 40 (5.7%) participants were not included. The major reasons for participants who were not included in the final analysis were due to death-9 (2.4%) from intervention and 8 (2.4%) from control groups, in total 17(2.4%); and due to transfer out-12 (3.3%) from intervention and 11 (3.3%) from control groups, in total 23(3.3%) [Fig 1]. Three (0.8%) participants from intervention group and 14(4.2%) participants from control group were lost to follow-up or they were defaulters. All defaulted participants were included in the final data analysis as non-adherent [Fig 1].

At baseline, approximately all participants’ characteristics were similarly distributed between intervention and control groups [Table 1]. Of total participants, 401 (57%) were male. Age range of participants was 18–90 years, and 457(66%) were less than 35 years old. One hundred and five (15%) of participants were at risk of alcohol use disorder, and 60(8.6%) were smokers at the time of study period. Almost half of participants 341 (49%) were with manifested mild to severe psychological distress at 16 cut-off point of K-10 items. The mean (±Standard deviation (Std)) score of psychological distress was 14 (±5) among intervention group and 19 (±8.1) among control group at endpoint of follow-up period. On ANCOVA analysis that was adjusted for baseline variables, there was significant difference in the mean score of knowledge level between control and intervention groups and similarly there was statistically significant difference in the score of each HBM domain between control and intervention groups at the endpoint (*p < 0*.*001*) [Table 2].

**Table 2:** Endpoint knowledge level and each HBM domains mean scores difference between intervention and control groups, adjusted for clusters.

Variable	Control		Intervention		*P-Value
	Mean (95% CI)	SD	Mean (95% CI)	SD	
Knowledge Level	30 (29–31)	4.8	35 (34–36)	2.6	< 0.001
Perceived Susceptibility	28 (27–29)	5.4	32 (31–33)	4.9	< 0.001
Perceived Severity	37 (36–38)	3.7	40 (40–41)	3.3	< 0.001
Perceived Barriers	60 (58–62)	12.5	52 (50–53)	10.9	< 0.001
Perceived Benefit	33 (32–34)	3.4	35 (34–35)	3.1	< 0.001
Cues to Action	32 (31–33)	3.9	34 (33–35)	6.5	< 0.001
Perceived Self Efficacy	51(50–52)	4.8	55 (54–55)	4.4	< 0.001

SD-Standard Deviation

*Cluster adjusted P-value

### Treatment Non-Adherence and Its HBM Domains Distribution

**Fig 2:**
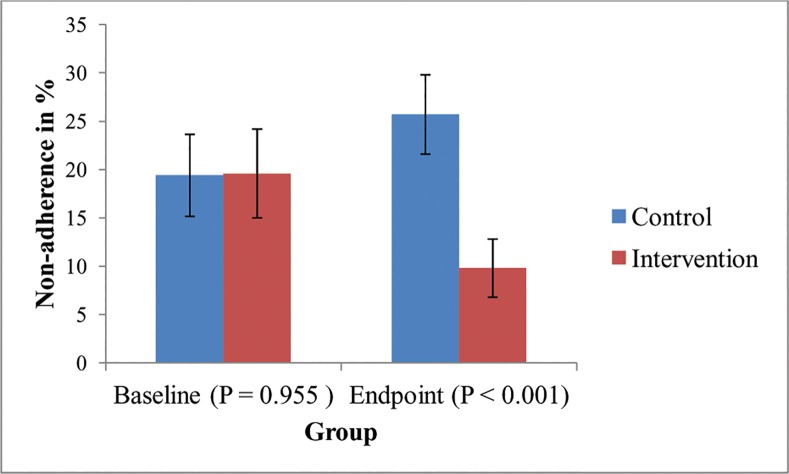
Non-adherence levels at baseline and endpoint among intervention and control groups (Figure shows % ± 95% CI, and cluster adjusted *p < 0*.*001*).

The overall non-adherence proportion was almost the same between intervention group (19.4%) and control (19.6%) group at baseline. However, at endpoint of study period, non-adherence among intervention group was 9.5%, while among control group was 25.4% (*p < 0*.*001*) [Fig 2].

**Fig 3:**
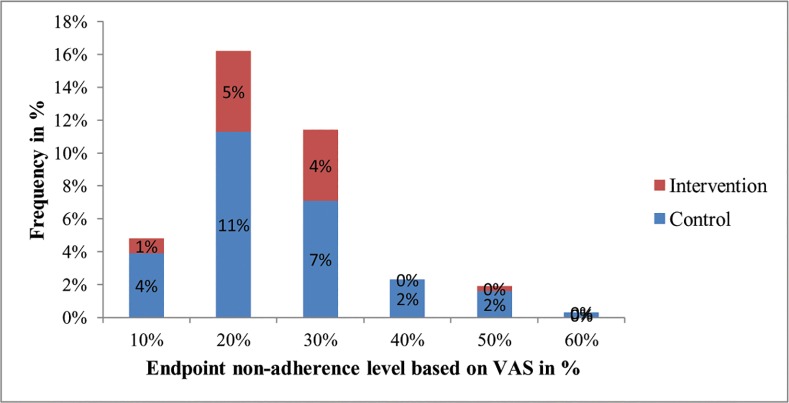
Endpoint non-adherence level distribution among control and intervention groups.

VAS was labeled as 0%, 10%, 20%, 30%, 40%, 50%, 60%, 70%, 80% 90% and 100% for assessing non-adherence level of participants [Fig 3]. Four percent of study participants were non-adherent among control group, while only 1% of them were non-adherent among intervention group at 10% of the scale. In addition, 11% of participants were non-adherent among control group and 5% were non-adherent among intervention group at 20% of VAS. Moreover, 7% of participants were non-adherent among control group, while 4% were non-adherent among intervention group at 30% VAS [Refer to Fig 3 for the detail].

**Fig 4:**
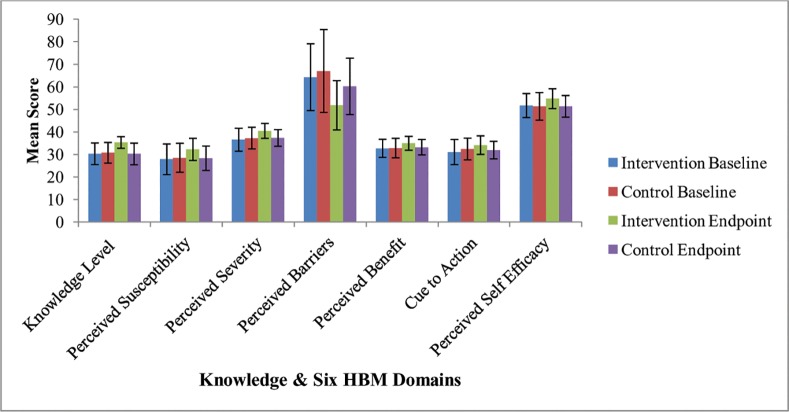
Baseline and endpoint mean scores difference of knowledge level and each HBM domains between control and intervention, adjusted for cluster (Figure shows Mean ± 95% CI).

At baseline, there was no statistical difference between adherent and non-adherent study participants with respect to knowledge level and the mean scores of each HBM domain. However, at endpoint of study period after adjusting for baseline variables and study clusters, there was significant difference between adherent and non-adherent study participants regarding the knowledge level and the mean score of each HBM domain (*p < 0*.*001*) [Fig 4].

### Results of Multilevel Model Analysis

Following psychological counseling and educational interventions, statistically significant difference was observed between the control and intervention groups with respect to non-adherence level, both in an unadjusted *(COR = 0*.*35*, *p <0*.*001*)) and adjusted (*AOR = 0*.*31*, *p < 0*.*001*) models [Table 3]. In an unadjusted multilevel logistic regression model, marital status (*COR = 1*.*7*, *p = 0*.*048*), ART status (*COR = 0*.*45*, *p = 0*.*024*), current smoking history (*COR = 2*.*3*, *p = 0*.*005*), alcohol use disorder (*COR = 2*.*5*, *p = 0*.*002*) and economic status (*COR = 0*.*91*, *p = 0*.*011*) were found to be significantly associated with treatment non-adherence. The overall multilevel logistic regression model consisted of all baseline HBM domains and other independent variables (included from unadjusted model) with p value less than 0.2 was statistically significant by likelihood ratio test (*p = 0*.*01*). In addition, in multilevel logistic regression analysis the variance (SE) of random effect was 0.28 (0.19) for model containing all HBM domains, and 0.29 (0.19) for model containing only HBM domain with p < 0.2 on Univariate analysis i.e. perceived self-efficacy [Table 3]. Finally, adjusted multilevel logistic regression analysis revealed that marital status (*AOR = 1*.*8*, *p = 0*.*051*), ART status (*AOR = 0*.*46*, *p = 0*.*011*), AUD (*AOR = 2*.*0*, *p = 0*.*033*) and psychological distress (*AOR = 0*.*97*, *p = 0*.*053)* were significantly predicted treatment non-adherence [Table 3].

**Table 3:** Crude and Adjusted Odds Ratios of Predictors of TB Treatment Non-adherence.

Characteristics		Unadjusted Multilevel Model ^a^		Adjusted Multilevel Model		Adjusted Multilevel Model ^b^	
		COR (95% CI)	P	AOR (95% CI)	P	AOR (95% CI)	P
Study Group	Control	1.00	< 0.001	1.00	< 0.001	1.00	< 0.001
	Intervention	0.35 (0.21–0.58)		0.31 (0.18–0.51)		0.33 (0.19–0.57)	
Gender	Female	1.00	0.084	1.00	0.539	1.00	0.477
	Male	1.5 (0.94–2.3)		1.2 (0.71–1.9)		1.2 (0.73–1.9)	
Marital Status	Married	1.00	0.029	1.00	0.051	1.00	0.044
	Unmarried	1.7 (1.1–2.9)		1.8 (1.00–3.1)		1.2 (1.0–2.9)	
ART Status	Not on ART	1.00	0.028	1.00	0.011	1.00	0.014
	On ART	0.45 (0.22–0.92)		0.46 (0.17–0.81)		0.39 (0.18–0.83)	
Current Smoking History	Non-smoker	1.00	0.010	1.00	0.084	1.00	0.087
	Smoker	2.3 (1.2–4.3)		2.0 (0.91–4.2)		1.9 (0.91–4.1)	
AUD Risk	No Risk	1.00	< 0.001	1.00	0.033	1.00	0.039
	There is risk	2.5 (1.5–4.2)		2.0 (1.05–3.9)		2.0 (1.0–3.7)	
Age (in year)		1.0 (0.98–1.0)	0.617		0.928		
Economic Status		0.91 (0.83–1.0)	0.043	0.93 (0.85–1.0)	0.168	0.93 (0.84–1.0)	0.136
Psychological Distress		0.97 (0.95–1.0)	0.083	0.97 (0.94–1.0)	0.053	0.97 (0.99–1.1)	0.034
Knowledge level		1.0 (0.97–1.1)	0.358	1.0 (0.98–1.1)	0.227	1.0 (0.94–1.0)	0.149
Perceived Susceptibility		1.0 (0.97–1.0)	0.814	1.0 (0.97–1.1)	0.634		
Perceived Severity		1.0 (0.95–1.0)	0.820	1.0 (0.94–1.1)	0.863		
Perceived Barrier		1.0 (0.98–1.0)	0.568	0.99 (0.98–1.0)	0.453		
Perceived Benefit		0.99 (0.94–1.0)	0.590	0.99 (0.92–1.1)	0.871		
Cue to Action		0.98 (0.94–1.0)	0.341	0.98 (0.92–1.0)	0.391		
Perceived Self-efficacy		0.97 (0.93 1.0)	0.114	0.97 (0.92–1.0)	0.251	0.96 (0.92–1.00)	0.051
		Variance (SE)		Variance (SE)		Variance (SE)	
		*		0.28 (0.19)		0.29 (0.19)	

* The center effects for each unadjusted multilevel models were different and not presented (because, for each variable the model was different).

^a^ Unadjusted multilevel model, but adjusted for cluster.

^b^ Adjusted Multiple Model after four HBM domains reduced from model.

## Discussion

Psychological counseling and adherence education interventions that were based on HBM concept significantly reduced treatment non-adherence level among intervention group. Our adjusted multilevel logistic regression model revealed that TB patients in intervention group were 0.31 times more likely to be non-adherent compared to patients in the control group, which suggested that our intervention decreased the chance of being non-adherent by 69%. In addition, being unmarried, availability of AUD and having psychological distress symptom were found to increase the chances of being non-adherent, while being on ART was found to have the effect of lowering the level of non-adherence behavior.

Several interventional studies have been conducted, aiming at decreasing the non-adherence level and their results are diverse and some are conflicting [24,25,44–48]. Although, some of these interventional studies used various methods, none of them used social science theoretical model guide for their interventions. That is why our intervention used HBM, targeting psychological distress and knowledge level among TB patients, so as to eventually reduce the non-adherence level. Study conducted by Kaliakbarova et al. [24] revealed that psychosocial support provided to MDR-TB patients was able to decrease treatment non-adherence level significantly. Moreover, Lee et al. [25] reported that physician education was able to decrease cumulative non-adherence level among intervention group significantly compared with the control group. Thiam et al. [48] also reported an improvement of TB treatment adherence level after intervention was directed focusing on “counseling through improved communication between health care workers and patients”. According to a systematic review reported by M’Imunya et al. [46], although the magnitude of benefits vary depending on intervention implemented, education or counseling intervention was able to increase adherence level. However, Liefooghe et al. [44] reported that intensive patient counseling had a limited impact on adherence improvement. In addition, study conducted by Morisky et al. [45] shown, intensive education and a monetary incentive could not improve significantly TB treatment adherence among intervention group. In our study, psychological counseling and health education interventions significantly decreased non-adherence level among intervention group. Moreover, unlike those studies that reported contradictory results, our findings were consistent with many other studies’ findings. In our view, the major reasons that the contradictions observed between some of previous studies’ results stated above and our study could be due to the quality of interventions implemented and the differences in contents of those interventions.

Previous studies conducted in Ethiopia showed that majority of TB patients who begin their anti-TB treatment as per the routine DOT strategy (without any intervention) tend to default and intermittently interrupt more during the continuation phase than the intensive phase [9–11]. This increases the non-adherence level among TB patients who are on DOT, and hence the increase in non-adherence level from 19.4% (at baseline) to 25.4% (at end point) among control group of our study is consistent with these studies [9–11].

Research evidence shows that, lack of knowledge about a particular disease and its treatment, distance from health care facility, perceived stigma, perception about disease and its treatment, psychological distress, change of residential place and economic status were some of several factors that determine TB treatment non-adherence [9–22]. Similarly, in our study marital status, ART status, AUD and psychological distress symptom were the factors that were predicted treatment non-adherence significantly.

Although psychological distress management is the most neglected area under DOTS, it is the most contributor of morbidity occurs with TB infection and leading to treatment non-adherence. For instance, according to Pachi et.al [16] systematic review report, psychological morbidity and other factors affect treatment adherence behavior of TB patients. This result was consistent with our finding in which TB patients who manifested psychological distress symptoms were at greater risk of being non-adherent than those TB patients without psychological distress. This may be due to psychological morbidity may lead to memory and decision impairment or maybe patients become hopeless and decide to abandon the treatment.

A qualitative studies reported from Ethiopia [12,13] demonstrated that being on dual treatment (on ant-TB and anti-HIV) was one of the major barriers of TB treatment adherence due to its drug burden and side effect of both drugs. This result contradicted with our findings in which being on ART decreased the chance of non-adherence by 63% among TB patients on dual treatment (anti TB and ART). These differences are most probably due to TB patients on dual treatment get more counseling services than those patients on anti-TB drugs alone.

Naidoo et al. [21] and Sendagire et al. [49] reported the existence of significant association between alcohol use disorder risk and TB treatment non-adherence. Moreover, according to a study reported from Uganda [50], most of TB patients who were drinking alcohol found to be residing in slam areas and these patients tend to give wrong addresses, and hence they would be easily lost to follow up and could not be traced back after interrupting their treatment. These studies’ results were in agreement with our study finding in which TB patients who had AUD on WHO AUDIT items were 2 times more likely to be non-adherent than those who had no AUD. This may be due to the fact that TB patients who drink alcohol may postpone taking medication to drink alcohol with friends, or they may miss their treatment due to memory and decision impairment resulting from alcohol use.

Social support, especially family support is very important for TB patients on treatment. According to previous studies, TB patients who are on treatment tend to interrupt their treatment due to lack of family and community support [12,13]. Family support is particularly quite useful for patients on TB treatment for psychological and financial support reasons [12,13]. This finding was consistent with our finding in which unmarried patients were 1.8 times more likely to be non-adherent than those who were married.

At endpoint of our study period, there was significant difference between intervention and control group with regard to the mean knowledge level and mean scores of each HBM domains (adjusted for baseline). Similarly, at endpoint (after adjusting for baseline and study cluster), there was significant difference between adherent and non-adherent patients regarding the mean knowledge level and the mean scores of each HBM domain. This shows that our intervention had an effect on patients’ knowledge level and health beliefs concerning TB disease and its treatment adherence among intervention group. Moreover, psychological counseling and educational intervention implemented based on HBM concept to enhance TB treatment could improve patients’ knowledge level and their health perceptions. Although, we couldn’t find sufficient interventional studies which applied HBM domains to enhance TB treatment adherence, a meta-analysis reported by DiMatteo et al. [21] showed a significant association between perceived seriousness of TB disease and higher level of treatment adherence among TB patients. This result was consistent with our finding in which patients’ health beliefs had significant impact on treatment non-adherence among intervention group.

The main limitation of this study was that transferred out patients were not traced. Therefore, in future, intervention targeting transferred out and designing strategies by which transferred out participants could be traced is vital. In addition, due to the budget limitation the interventions provided were once per month, which may not be adequate, and hence this might have resulted in minimal improvement of mean score changes of knowledge level, psychological distress and mean score of each HBM domain among intervention group. Moreover, health professionals working at each TB clinic were made to administer the data collecting instrument and also they were the ones who implemented the interventions. Health professionals might have under reported the non-adherence level due to fear of being noticed by their supervisors as low performers. On the other hand, participants themselves might have underreported the barriers related to health care facilities and health care workers, as the interviews themselves were health workers serving participants. Hence, the potential biases were not only in one direction, rather it could be overestimation or under estimation of the non-adherence level. Therefore, despite the limitations stated above, we do believe that results were less likely to be influenced by those limitations.

## Conclusion

Our psychological counseling and adherence education intervention resulted in decline of non-adherence level significantly among intervention group at endpoint of the follow up period. Therefore, to decrease the level of non-adherence among TB patients on treatment, provision of psychological counseling and health education services to TB patients is recommended. Furthermore, as part of the strategy of TB treatment, incorporating psychological counseling and health education services into conventional DOTS strategy is recommended.

## Supporting Information

S1 CONSORT Checklist (DOC)

S1 Protocol (PDF)
