# Supplementary material for: Psychological and Educational Intervention to Improve Tuberculosis Treatment Adherence in Ethiopia Based on Health Belief Model: A Cluster Randomized Control Trial
Source: PLoS One. 2016 May 11;11(5):e0155147. doi: 10.1371/journal.pone.0155147 (PMC4864292; doi:10.1371/journal.pone.0155147)

Tehran University of Medical Sciences

International Campus

School of Public Health

A Research Proposal for Partial Fulfillment of MSc Degree in Health Education and Promotion Submitted to Tehran University of Medical Sciences, International Campus (TUMS-IC) office of the Deputy for Research Affairs

Psychosocial and Behavioural Determinants of TB Treatment Non-adherence, and Planning Educational Intervention to Improve Treatment Adherence in Ethiopia: Guided by Health Belief Model

By

Habteyes Hailu Tola

Supervisor: Dr Davousd Shojaeizadeh (PhD, Full professor)

Advisors:

1. Dr Gholamreza Garmarodi (MD, PhD, Associate professor)
2. Dr Azar Tol (PhD, MPH)
3. Dr Mir Saeed Yekaninejad (PhD , Assistant Professor of Biostatistics)

February, 2014, Tehran, Iran

RESEARCH ETHICS APPROVAL SUBMISSION FORM

Project No. 9113422001 (given by Committee)

<table>
<tr><td colspan="4">Title: Psychosocial and Behavioural Determinants of TB Treatment Non-adherence, and Planning Educational and Psychosocial Intervention for Treatment Adherence in Ethiopia: Guided by HBM</td></tr>
<tr><td colspan="4">Person (s) undertaking the project (in order of contribution)</td></tr>
<tr><td>Name</td><td>Qualification</td><td>Institution/ Company</td><td>Country</td></tr>
<tr><td>Habteyes Hailu Tola (PI)</td><td>MSc student in Health Education and Promotion</td><td>TUMS/EPHI</td><td>Ethiopia/Iran</td></tr>
<tr><td>Davoud Shojaeizadeh</td><td>PhD in Health Education and Promotion (Full Professor)</td><td>TUMS</td><td>Iran</td></tr>
<tr><td>Azar Tol</td><td>PhD (MPH) in Health Education and Promotion</td><td>TUMS</td><td>Iran</td></tr>
<tr><td>Gholamreza Garmarodi</td><td>MD, PhD, MPH, (Assistance Professor)</td><td>TUMS</td><td>Iran</td></tr>
<tr><td>Mir Saeed Yekaninejad</td><td>PhD in Biostatistics (Assistance Professor)</td><td>TUMS</td><td>Iran</td></tr>
<tr><td>Gebremedhin G/ Mechael</td><td>MSc student in Infectious Disease</td><td>EPHI</td><td>Ethiopia</td></tr>
<tr><td>Abebaw Kebede</td><td>MSc in Infectious Disease</td><td>EPHI</td><td>Ethiopia</td></tr>
<tr><td colspan="2">Source of budget: Under process</td><td colspan="2">Total cost of the project 7,013.60 $</td></tr>
<tr><td>Study Period</td><td colspan="2">Date of commencement: May,01/ 2014</td><td>Date of completion: November, 30/ 2014</td></tr>
<tr><td colspan="4">Institution at which study to be conducted: EPHI and Tehran University of Medical Sciences
Habteyes Hailu Tola: Cell No: +251 913006836/+989336449784/, Email: habtetola@gmail.com</td></tr>
</table>

Abbreviations and Acronyms

AIDS	Acquired Immunodeficiency Syndrome
ART	Antiretroviral Therapy
AUDIT	Alcohol Use Disorder Identification Test
CMD	Common Mental Disorder
DOT	Direct Observation of Therapy
DOTS	Directly Observed Short-course Therapy
EPHI	Ethiopian Health and Nutrition Research Institute
HBM	Health Belief Model
HC	Health Center
HIV	Human Immunodeficiency Virus
MDR-TB	Multi Drug Resistant TB
RECC	Research Ethical Clearance Committee
RERB	Research Ethical Review Board
SPSS	Statistical Package for Social Sciences
TB	Tuberculosis
TUMS	Tehran University of Medical Sciences
TUMS-IC	Tehran University of Medical Sciences International Campus
VAS	Visual Analogues Scale
WHO	World Health Organization

Abstract

This study intends to determine psychosocial and behavioral determinants of TB treatment non-adherence, and to evaluate psychological and educational intervention to improve treatment adherence among TB patients on treatment based on Heath Belief Model (HBM) concept. HBM is recommended as effective and useful model to understand and explain health behavior including treatment adherence as healthy practice by patients. It is a psychological model which constructs from six domains. These domains are perceive susceptibility to and perceived severity of the condition, perceived benefits of the recommended behavior, perceived psychological/ tangible barriers to perform recommended behavior, cue to action (motivator) to perform the behavior and self- efficacy on performing the recommended behavior correctly. Patients in treatment are likely adheres to their medical regimen under specific five sets of conditions according to the concept of HBM. First, patients must have some minimal health knowledge and motivation towards staying free of TB. Second, patients must perceive themselves as vulnerable to TB disease and they must also believe that TB and consequences of non-adherence are clearly a serious medical and health problem. Third, patients must also be convinced that current treatment TB is effective, that it is indeed possible to obtain control over the problems at an acceptable psychological or tangible social, psychological barriers and that the barrier does not outweigh the benefits. Fourth, the presence of an internal or external stimulus, referred as “cue to action,” the trigger the health behavior of patients such as taking medication regularly. Finally, patients’ self-efficacy belief on performing the recommended behavior should be maintained till the final treatment period. Therefore, based on HBM, the main outcome variable planned to be measured is level of treatment non-adherence. The key independent variables that are thought to be influencing the outcome variable and that will be assessed are six HBM domains; and as the main influencing factors, psychological variables (depression, anxiety and fear of stigma), social factors (demographic variables, economic problems and social support), and behavioural factors (knowledge, feeling better after few weeks of treatment, alcohol and tobacco use).

The cluster randomized control trial preceded by cross sectional study will be conducted in Addis Ababa from March to October, 2014 at selected health care facilities. Three hundred forty two (342) eligible TB patients will be enrolled consecutively from randomly selected 30 health centers which selected by random cluster sampling method. The health centers will be randomly allocated into control and intervention group. Researcher developed and standard questionnaires will be used for data collection after validated for reliability. The questionnaire will be administered by trained health professional at TB clinic of each health centers at baseline and endpoint. After, baseline information collected psychological and educational intervention will be implemented for intervention group, while control group followed under conventional TB treatment strategy. At the end of follow up similar data will be collected from both groups. Association factors and the impact of intervention will be assessed by multiple logistic regression, at 5% level of precision using SPSS ver.21.0.

Ethical approval will be obtained from the research ethical review board, Tehran University of Medical Sciences, Research and ethical review committee of Ethiopian Public Health Institute and Addis Ababa Health Bureau. Both oral and written informed consent will be obtained from each study participant. The experience and information gained from this study should help to improve TB prevention and control program; and to enhance TB treatment adherence.

1. Background

Adherence to long term treatment like Tuberculosis (TB) and Human Immunodeficiency Virus (HIV) is very challenging because of its long term and drug related side effects [1]. As known treatment adherence is very important for effective treatment outcome and prevention of drug resistant TB bacilli strain. However, TB treatment non-adherence has several socioeconomic and health consequences [1-5]. From several consequences, occurrence of multi drug resistant TB (MDR-TB) is one of serious consequence that challenging global TB control program. For instance the odds of having interrupted the treatment at least for one day among those who developed (MDR-TB) are 13 times than among those not interrupted at all [6].

According to evidences, several psychosocial and behavioral factors are responsible for TB treatment non-adherence. For instance, depression [7], fear of stigma [8] and individual negative emotional condition [9] are the main psychological factors that associated with TB treatment non-adherence. In addition, lack of transportation cost and food, lack of social support, unemployment, lack of permission from job place and lack of shelter are some of social factors that influence TB treatment non-adherence [10-15]. Beside psychosocial factors, individual behavioral factors like individual TB disease and its treatment knowledge, feeling better after few weeks of treatment and forgetfulness are factors associated with treatment non-adherence [15-18]. Furthermore, alcohol consumption and tobacco smoking are the two individual behavioral factors that associated with TB treatment non-adherence [12, 17, 19-20].

Beside psychosocial and behavioural factors lack of support from health care workers, and health care worker poor communication, distance from health care centre, insufficient and interrupted TB medication and laboratory logistic supply are health care worker and facility related factors that associated with TB treatment non-adherence [10, 11, 14]. In addition, TB patient's HIV and anti retroviral therapy (ART) status are association with TB treatment non-adherence [11].

However, contrarily other studies are reported that HIV sero positive and on ART are preventive factors of TB treatment non-adherence and lost to follow up among TB patients with HIV [11, 14]. In order to improve TB treatment adherence level, various interventions designed and implemented across the globe [21-23]. For instance, an intervention conducted based on enhanced TB adherence model, which focused on patient and professional empowerment, proved to be positively impacting TB treatment adherence [21]. Moreover, a study conducted in East Kazakhstan shows that psychosocial support for MDR-TB patients improves treatment adherence [22]. On the other hand, educational intervention that was provided by physician in Bangladesh was proven to increase the cumulative adherence level among intervention group than control group [23]. However, these all interventions are limited to particular sociocultural settings and these interventions need to be replicated in different areas.

Although evidences suggest public health and health promotion interventions based on social and behavioral science theories are more effective than those without theoretical model base [24, 25], available interventional studies related to TB treatment adherence lack health behavioral theoretical model base. Moreover, there are no considerable interventions that implemented to assess the applicability of theoretical models for TB treatment adherence promotion through targeting factors influence patients' adherence behavior at specific sociocultural context. Ethiopia is one of those countries where such information is lacking despite the fact that TB treatment non-adherence level is high (ranging from 10% to 21%) [26-28], and the prevalence of MDR-TB is also increasing from 1.6% to 2.3% among new cases and from 12% to 17.8% among previously treated cases [29, 30]. Hence, determining psychosocial and behavioral predictors of TB treatment non-adherence, and evaluating of interventional experiences based on theoretical model in particular sociocultural context to promote TB treatment adherence is essential. Therefore, this study was aimed to determine psychosocial and behavioural predictors of TB

treatment non-adherence, and to evaluate a combined psychological counseling and educational intervention to enhance TB treatment adherence in Ethiopian based on HBM.

HBM is recommended as effective and useful model to understand, explain and predict health behavior of individuals including treatment adherence as healthy practice by patients [31-33]. It is a psychological model that constricts from six domains [34, Fig 1]. These domains are perceived susceptibility, perceived severity, perceived benefit, perceived barriers, cue to action and perceived self-efficacy. According to HBM concept, TB patients on treatment are likely adhere their medical regimen under specific five sets. First, patients must have some minimal health knowledge and motivation towards staying free of TB disease. Second, patients must perceive themselves as vulnerable to TB disease and they must also believe that TB disease and the consequences of non-adherence is clearly a serious medical and health problem. Third, patients must also be convinced that current TB treatment is effective, that it is indeed possible to obtain control over the problems at an acceptable psychological or tangible social barriers and that the barrier does not outweigh the benefits. Fourth, the presence of an internal or external stimulus, referred as "cue to action," the trigger the health behavior of patients such as taking medication. Finally, patients' self-efficacy belief on regular follow up of treatment should be maintained till the final treatment period. Figure 1 shows the relationship between six HBM domains and individual participants baseline characteristics.

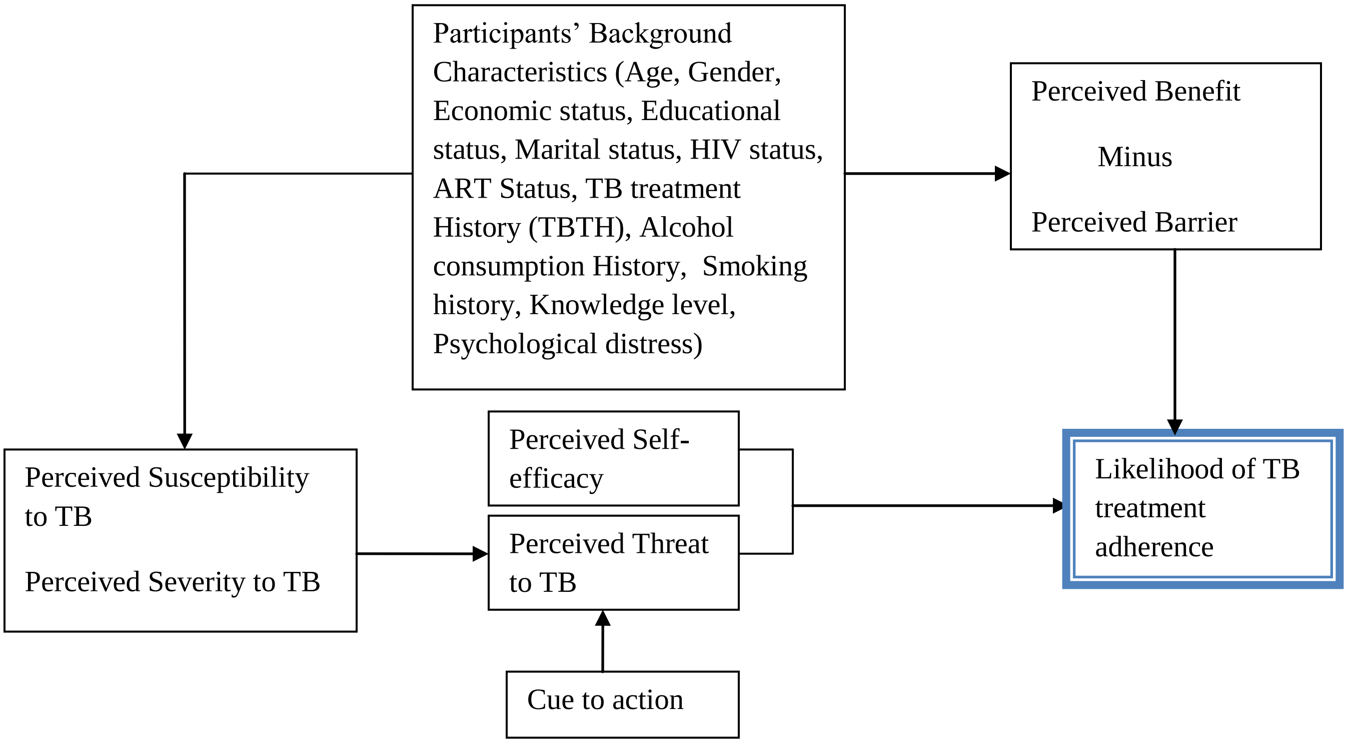

Figure 1: Health Belief Model and TB treatment adherence adopted from Becker MH (1974) Health Belief Model

2. Objectives

2.1. General Objective:

To determine psychosocial and behavioural predictors of TB treatment non-adherence, and to evaluate a combined psychological counseling and educational intervention to enhance TB treatment adherence in Ethiopian based on HBM.

2.2. Specific Objectives

1. To describe frequency distribution of TB treatment non-adherence in terms of age, gender, marital status, educational status, economic status, employment condition, marital status, HIV status, antiretroviral status, TB treatment history, TB type, smoking history, alcohol use history, tobacco smoking history, psychological distress and distance from treatment center .
2. To determine TB treatment non-adherence level difference between intervention and control groups
3. To determine psychosocial and behavioral predictors of TB treatment non-adherence.
4. To determine the impact of psychological and educational intervention on TB treatment non-adherence
5. To determine the mean difference between intervention and control groups on six HBM domains

3. Materials and Methodology

3.1. Implementation of Study

A Cluster Randomized Control Trial (RCT) preceded by cross sectional study will be conducted among all forms of TB patients at selected primary health care facilities in Addis Ababa from May to November, 2014. Cross-sectional study at baseline will be conducted with the aim of identifying the baseline information of participants on treatment non-adherence based on HBM six domains which are well explained in background part of this document and clearly shown by figure 1. Three hundred forty two (342) TB patients on treatment under DOTS strategy for one to two month of treatment initiation will be enrolled for each group (control and intervention) at selected primary health care facilities. To achieve this sample size, 30 health centers will be selected by random cluster sampling, and divided into two equal groups (15 for control and 15 for intervention) by simple random sampling technique. Randomizing of health facility is to control socioeconomic differences of each sub-city, and to control information contamination between control and intervention group on educational and psychosocial intervention will be applied. Participants will be enrolled from health center selected as a control will be control group and participants recruited from health center selected as intervention group will be intervention group of the study. Also, we assume, as we will be able to enroll at least one participant per day at each selected primary health care facility to achieve our target sample size (34) at single health center.

Researcher designed and standardized questionnaire will be used for data collection after evaluating for validity by a group of experts and for reliability by pilot study at study area. Then baseline/cross-sectional study data on psychosocial and behavioural determinants of TB treatment non-adherence will be collected thoroughly.

The intended psychological and educational intervention will be provided every week for the first month of enrollment, every two weeks during the second month, and then every month until the end of DOTS treatment for drug susceptible type of TB patients. Implementation of intervention will be based on experience obtained from other studies such as study reported from Central Asian region [35, Kazakhstan [22], and Bangladesh [23]. However, our study is slightly different from these previous studies, because it is based on the concept of HBM.

At the end of treatment completion, similar questionnaire with baseline will be applied to both groups, and the endpoint data will be collected to see the impact intervention.

Data collected under intensive supervision of principal investigator and other members of the study team will be entered into double spread sheet data bases, checked for quality and will be analyzed with Statistical Package for Social Sciences (SPSS) version 21.0 at 5% precision level; and the result will be disseminated through oral presentation on different events and publication on peer reviewed journal.

3.2. Study Population

Study population is all TB patients who are diagnosed for all types of TB based on national TB treatment guideline and on first line and MDR-TB treatment regimens under DOTS strategy and MDR-TB treatment protocol [36]. Treatment adherence will be considered as the extent to which the patient's history of therapeutic medication-taking coincides with the prescribed treatment [1]. Accordingly, we will consider treatment adherent patient as a patient's attendance at the scheduled visit and regular medication with over 90% of doses prescribed based on WHO adherence definition [1].

3.3. Inclusion and Exclusion Criteria

3.3.1. Inclusion criteria

1. TB patients 18 years or older
2. TB patients on full course of TB treatment for at least one month prior to the study and under normal DOTS strategy
3. TB patients who are physically, mentally capable to provide informed consent, and can follow intervention provided without any burden or support
4. TB patients who willing to participate in the study

3.3.2. Exclusion

1. TB patients who are participating in other interventional studies
2. TB patients who are not on full course of treatment under DOTS therapy

3.4. Sampling and Sample Size Determination

As clearly demonstrated in figure 2 bellow; from 53 health center found in Addis Ababa, we will select 30 health centers from 10 sub-city administrations by random cluster sampling method. Then 30 health centers will be divided into 15 control and 15 intervention groups by simple random assignment while considering control and interventional sites are far from each other to prevent information contamination.

To estimate précis proportion of non-adherence level; TB treatment non-adherence determinant factors; and the mean deference of two groups (control and interventional groups) on treatment non-adherence will be used two population proportion estimation formula to determine the required sample size.

$$n = \frac{(Z_{\alpha/2}+Z_{\beta})^2 \ X \ (p_1(1-p_1) + p_2(1-p_2))}{(p_1 - p_2)^2}$$

Where $Z_{\alpha/2}$ is the critical value of the normal distribution at $\alpha/2$, Z_β is the critical value of the normal distribution at β, p_1 and p_2 are the expected sample proportions of the two groups. By taking p_1 = 79% (0.79) proportion of TB patients are adherent for their treatment according to previously reported result [26] under normal TB treatment strategy (DOTS), and by hypothesizing p_2 = 89% (0.89) our intervention will increase TB treatment adherence at least by 10% among interventional group, and by considering 95% confidence interval, 80% power, 5% margin of error and equal sample size for each groups. Then, the sample size will be:

$$n = \frac{(1.96+0.84)^2 \ X \ [0.79(1-0.79) + 0.89(1-0.89)]}{[0.89-0.79]^2}$$

20681.92/100 = 206.82 ≈ **207** for each groups

Because, our study is cluster randomized trial, we assumed design effect 1.5 by cluster sampling method to overcome the design effect. Then, the sample size will be:

207 X 1.5 = 3110.5 ≈ 311

Finally, sample size further increased by 10% to account for contingencies such as non-response or recording error, i.e. 311 X 10/100 + 311= 3342.1 ≈ **342.** Therefore, the final sample size will be **342** TB patients on first line treatment for each group. As clearly shown in figure 2 bellow, we will be divided 2*342 participants into equal 34 participants at 20 each health center.

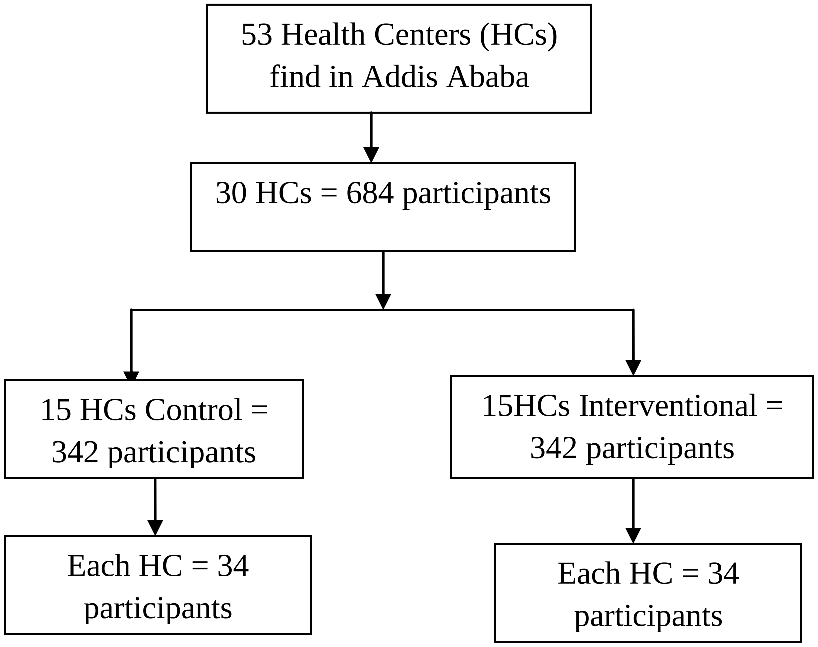

Fig 2: Sampling method for first line medication susceptible TB participants

3.5. Instrument and Data Collection

Kessler-10 Psychological Distress Scale (37), WHO 10-items Alcohol Use Disorder Identification Test (AUDIT) (38) and Government of Western Australian, Mental Health Smoking Assessment Check list (39) are standard questionnaires will be used to collect data for psychological disorder, alcohol consumption and tobacco smoking respectively. The cutoff point will be considered according to standard questionnaire guideline and previous similar studies. For socio-demographic data and participants' TB disease and its treatment knowledge level related information and adherence level literature based designed questionnaire will be used. Health professional at TB clinic of selected health facilities will be applied the questionnaire after 4 days intensive training on questionnaire and good clinical practice. Finally, educational and psychological intervention will be provided by appropriate professional according scheduled.

3.6. Data Entry and Analysis

Data will be double interred in standard spreadsheets separately for quality purpose. Then, it will be analyzed using Statistical Package for Social Sciences (SPSS) version 20.0. Descriptive information will be determined to describe the socio-demographic data. Data will be checked for normality and homogeneity distribution before assessment of association by using Kolmogorov-Smirnov or Shapiro-Wilk.

Associations between psychosocial and behavioral factors, HBM constructs (perceived susceptibility, perceived severity, perceived benefits, perceived barriers, cue to action and perceived self-efficacy) and TB medication non-adherence will be assessed by regression analysis at level of precision 5%, i.e. *P* value less than 0.05 will be considered as significant.

3.7. Data Collection Management and Safety Consideration

As we were tried to explain in the sampling method, health facility for control and interventional groups will be selected randomly while considering the optimum distance between control and intervention group to prevent information contamination and to distribute socioeconomic variables difference between sub-cities. The instrument designed based on literature review will be approved for validity and reliability. Psychological and educational intervention will be designed by the research team who are professional and experienced in the field of health education and promotion and applied by thoroughly trained appropriate professionals. Data collection will be supervised and checked before electronic data entry by principal investigator and other study team member closely to assure the maximum quality level. Then, double data entry method will be employed in separated spread sheet and data accessibility will be restricted to data manager and study principal investigator. Before analysis, data will be check for normality and homogeneity, then analyzed & interpreted with health education and promotion

expert and senior biostatistician. Finally, quality scientific report will be developed and disseminated.

3.8. Ethical Consideration

Ethical approval will be obtained from the research ethical review board (RERB), Tehran University of Medical Sciences (TUMS), the Research and ethical review committee (RECC) of Ethiopian Public Health Institute (EPHI) and Addis Ababa City Administration Health Bureau. Both oral and written informed consent will obtain from each study participant after thoroughly explaining objectives and benefits of the study. To ensure confidentiality, any personal identifying information on participants will not be collected. Benefits, compensation mechanism will be strictly respected for all participants equally for the time they will expend with us during interview and intervention implementation. Data will be collected for this study will not be used for other study without approval of each participant. All international and institutional randomize control trial research ethics conventions will be strictly obeyed during our study process.

3.9. Benefits of the Study Results

The benefits of this study are clear; because it will generate comprehensive information on determinants of TB treatment non-adherence and evaluate the intervention for implementation to minimize TB treatment non-adherence, which is the main challenge for global TB control program. Therefore, it will be helpful in terms of treatment cost reduction, policy direction and quality life improvement for the patients. In addition, the implementation of study result will be simple, because it implemented alongside with previously in system TB treatment strategy (DOTS).

4. Implementation of the Study by Phases

S/No	Activities by phases for both drug susceptible and MDR types of TB		Time frame
1	**Phase -I**		**February- May 2014**
1.1	Protocol submission to TUMS RERB and EPHI RECC and Addis Ababa Health Bureau and obtain Ethical approval.		
1.2	Applying for fund		
1.3	Recruitment data collectors		
1.4	Training for data collectors and study staff members		
1.5	Letter of cooperation to selected health care facilities and to any concerned bodies		
1.6	Preliminary site assessment and arrangement		
1.7	Pilot study for instrument evaluation for validity and reliability		
2	**Phase - II**		
2.1	Participant recruitment, base line data collection and having an appointment with participants		June,1- 30/2014
2.2	Interpretation package designing		July,01- 30/2014
3	**Phase -III**		01/ 07-21/08/2014
3.1	Intervention implementation		
3.2	Month 1	Session 1	01/07/2014
		Session2	08/07/2014
		Session 3	15/07/2014
		Session 4	22/07/2014
3.3	Month 2	Session 1	01/08/2014
		Session 2	15/08/2014
3.4	Month 3	Session 1	01/09/2014

S/No	Activities by phases for both drug susceptible and MDR types of TB		Time frame
3.5	Month 4	Session 1	01/10/2014
3.6	Endpoint data collection from both groups		01/11/2014
4	**Phase - IV**		22/11/-30/012/2014
4.1	Data entry and analysis		
4.2	Report writing and dissemination		

5. Budget Break of the Study

S/No	Items	Cost for drug susceptible TB in $
1	Stationary (pen, pencil, note book, print paper and printer toner)	285.00
2	Plastic bag/folder for data collectors	60.00
3	Patient compensation	3510.00
	Transportation	432.00
4	**Personnel cost**	
	Intervention package designing personnel	426.00
	Intervention provider and data collector	421.00
5	Supervision	321.00
6	Staff training	695.00
7	Communication	226.00
**Total**		**6,376.00**
**Contingency (10%)**		**637.60**
**Grand Total**		**7,013.60 $**

6. References

1. WHO (2003). Adherence to long-term therapies: Evidence for action. WHO press, Geneva, Switzerland
2. Boogaard J Van Den, Boeree MJ, Kibiki GS, Aarnoutse RE. The complexity of the adherence-response relationship in tuberculosis treatment : why are we still in the dark and how can we get out ? *J. Tro. Med. and Inter. Health*, 2011;16(6):693–8.
3. Payero MÁ, Castro NML De, Samartín MU, Vila AM, López CV, Piñeiro G. Medication non- adherence as a cause of hospital admissions. *Farm Hosp*; 2014;38(4):328–33.
4. Hovstadius B, Petersson G. Non-adherence to drug therapy and drug acquisition costs in a national population - a patient-based register study. *BioMed Central Ltd*; 2011;11(1):326.
5. Pettit AC, Cummins J, Kaltenbach LA, Sterling TR Warkentin JV. Non-adherence and drug-related interruptions are risk factors for delays in completion of treatment for tuberculosis. *Int J Tuberc Lung Dis*. 2013;17(4):486–92.
6. Hirpa S, Medhin G, Girma B, Melese M, Mekonen A, Suarez P, Ameni G (2013), Determinants of multidrug-resistant tuberculosis in patients who underwent first-line treatment in Addis Ababa: a case control study, *BMC Public Health* (13):782
7. Pachi A, Bratis D, Moussas G, Tselebis A (2013), Psychiatric Morbidity and Other Factors Affecting Treatment Adherence in Pulmonary Tuberculosis Patients, Hindawi Publishing Corporation, *Tuberculosis Research and Treatment*, Vol. 2013, Article ID 489865
8. Kaliakbarova G, Pak S, Zhaksylykova N, Raimova G, Temerbekova B, van den Hof S (2013), Psychosocial Support Improves Treatment Adherence Among MDR-TB Patients: Experience from East Kazakhstan, *The Open Infe. Dis. J, 7, (Suppl 1: M7) 60-64*
9. Jakubowiak WM, Bogorodskaya EM, Borisov SE, Danilova ID, Lomakina OB, Kourbatova EV (2008), Impact of socio-psychological factors on treatment adherence of TB patients in Russia, *Tuberculosis* (Edinb) ;88(5):495-502. doi: 10.1016/j.tube.2008.03.004.
10. Sagbakken M, Frich C J, Bjune G (2008), Barriers and enablers in the management of tuberculosis treatment in Addis Ababa, Ethiopia: a qualitative study, *BMC Public*;, 8:11 doi:10.1186/1471-2458-8-11: available from: http://www.biomedcentral.com/1471-2458/8/11
11. Naidoo P, Peltzer K, Louw J, Matseke G, Mchunu G, Tutshana B (2013), Predictors of tuberculosis (TB) and antiretroviral (ARV) medication non-adherence in public primary care patients in South Africa: a cross sectional study, *BMC Public Health*, 13:396, *BioMed Central Ltd*: http://www.biomedcentral.com/1471-2458/13/396
12. Muture N B, Keraka N M, Kimuu K P, Kabiru W E, Ombeka O V, Oguya F (2011), Factors associated with default from treatment among tuberculosis patients in Nairobi province, Kenya: A case control study, *BMC Public Health*, 11:696, *BioMed Central Ltd*: http://www.biomedcentral.com/1471-2458/11/696
13. Maruza M, Albuquerque M FP, Coimbra1 I, V Moura L, R Montarroyos U (2011), Risk factors for default from tuberculosis treatment in HIV-infected individuals in the state of Pernambuco, Brazil: a prospective cohort study, *BMC Infectious Diseases*, 11:351, *BioMed Central Ltd*: http://www.biomedcentral.com/1471-2334/11/351
14. Tadesse T, Demissie M, Berhane Y, Kebede Y, Abebe M (2013), Long distance travelling and financial burdens discourage tuberculosis DOTs treatment initiation and compliance in Ethiopia: a qualitative study, *BMC Public Health*, 13:424, *BioMed Central Ltd*: http://www.biomedcentral.com/1471-2458/13/424
15. Gebremariam K M, A Bjune G, C Frich J (2010), Barriers and facilitators of adherence to TB treatment in patients on concomitant TB and HIV treatment: a qualitative study, *BMC Public Health*, 10:651, *BioMed Central Ltd.*: http://www.biomedcentral.com/1471-2458/10/651

16. Elbireer S, Guwatudde D, Mudiope P, Nabbuye-Sekandi J, Manabe C Y (2011), Tuberculosis treatment default among HIV-TB co-infected patients in urban Uganda, *Tropical Medicine and International Health* v.16 (8) pp 981–987 :doi:10.1111/j.1365-3156.2011.02800.x
17. Cramm M J, JM Finkenflüge H, Møller V, P Nieboer A (2010), TB treatment initiation and adherence in a South African community influenced more by perceptions than by knowledge of tuberculosis, *BMC Public Health*, 10:72, *BioMed Central Ltd*: http://www.biomedcentral.com/1471-2458/10/72
18. Castelnuovo B (2010), A review of compliance to anti tuberculosis treatment and risk factors for defaulting treatment in Sub Saharan Africa, *African Health Sciences*; 10(4): 320 - 324
19. Ayisi G J, H van't Hoog A, A Agaya J, Mchembere W, O Nyamthimba P (2011), Care seeking and attitudes towards treatment compliance by newly enrolled tuberculosis patients in the district treatment programme in rural western Kenya: a qualitative study, *BMC Public Health*, 11:515, *BioMed Central Ltd*: http://www.biomedcentral.com/1471-2458/11/515
20. Cramm M J, JM Finkenflüge H, Møller V, P Nieboer A (2010), TB treatment initiation and adherence in a South African community influenced more by perceptions than by knowledge of tuberculosis, *BMC Public Health*, 10:72, *BioMed Central Ltd*: http://www.biomedcentral.com/1471-2458/10/72
21. Atkins S. Improving Adherence: An evaluation of the enhanced tuberculosis adherence model in Cape Town, South Africa. *Publ by Karolinska Institutet*, Print by US –AB [Internet]. 2011; Available from: 978-91-7457-220-9
22. Kaliakbarova G, Pak S, Zhaksylykova N, Raimova G, Temerbekova B, Hof S Van Den. Psychosocial Support Improves Treatment Adherence Among MDR-TB Patients : Experience from East Kazakhstan. *The Open Infe. Dis. J, 7, (Suppl 1: M7,* 2013;60–4.
23. Lee S, Khan OF, Seo JH, Kim DY, Park K, Jung S et al. Impact of Physician ' s Education on Adherence to Tuberculosis Treatment for Patients of Low Socioeconomic Status in Bangladesh. *Chonnam Med J*, 2013; 49:27–30.
24. Glanz K, Bishop DB. The role of behavioral science theory in development and implementation of public health interventions. *Annu Rev Public Health*. 2010;31:399–418.
25. Munro S, Lewin S, Swart T, Volmink J. A review of health behaviour theories: how useful are these for developing interventions to promote long-term medication adherence for TB and HIV/AIDS? *BMC Public Health.* 2007;7:104.
26. Kebede A, Wabe T. Medication Adherence and its Determinants among Patients on Concomitant Tuberculosis and Antiretroviral Therapy in South West Ethiopia. *N Am J Med Sci*. 4AD;2:67–71.
27. Adane AA, Alene KA, Koye DN, Zeleke BM. Non-adherence to anti-tuberculosis treatment and determinant factors among patients with tuberculosis in northwest Ethiopia. *PLoS One* [Internet]. 2013 Jan [cited 2014 Sep 3];8(11):e78791.
28. Kiros YK, Teklu T, Desalegn F, Tesfay M, Klinkenberg E Mulugeta A. Adherence to anti-tuberculosis treatment in Tigray, Northern Ethiopia. *PHA.* 2014;4(4):S31–6.
29. Ethiopian Public Health Institute. Second Round National Anti-tuberculosis Drug Resistance Surveillance in Ethiopia Draft Report, EPHI TB-HIV Survey and related dissemination Workshop September 16-18, *Bahrdar, Ethiopia*. 2014.
30. Ethiopian Federal Minster of Health of Ethiopian/ Ethiopian Public Health Institute. First round TB drug resistant Surveillance report-Ethiopian. Addis Ababa, Ethiopia, 2005.
31. Redding CA, Rossi JS, Rossi SR, Velicer WF, Prochaska JO (2000). Health behaviour models. *Int Electr J Health Educ* (3):180-193

32. Llongo I (2004), Tuberculosis health belief gaps of tuberculosis and suspected tuberculosis cases in New York City, *Int J Clin Health Psychol.;* (4)1: 69-90
33. Barnhoon N F, Adriaanse H (1992). In search of factors responsible for non-compliance among tuberculosis patients in Wardha district, India. *Social Science and Medicine*, (34): 291-306.
34. Glanz K, Rimer BK, Viswanath K (2008), Health Behavior and Health Education: Theory, Research, and Practice 4th ed. Published by Jossey-Bass, USA, San Francisco
35. International Federation of Red Cross and Red Crescent Society (2013), Kyrgyzstan Consolidated Development Operational Report from 1 January 2013 to 30 June 2013
36. Ethiopian Federal Ministry of Health (2013), Guidelines for Clinical and Programmatic Management of TB, TB/HIV and Leprosy in Ethiopia, Fifth Edi. pp- 11, 14
37. Kessler R, Andrews G, Colpe LJ, Hiripi E, Mroczek DK, Normand S-LTet.al (2002), Short screening scales to monitor population prevalence and trends in nonspecific psychological distress, *Psychol Med*; 32:959. e976.
38. Babor T F, Higgins-Biddle J C, Saunders J B, Monteiro M G: WHO (2001), The Alcohol Use Disorders Identification Test Guidelines for Use in Primary Care, Second Edition, WHO, Department of Mental Health and Substance Dependence, Geneva, Switzerland
39. Government of Western Australian, Department of Health (2013): Mental Health Smoking Assessment Check list, HP012591 JAN’13: Online Available at http://www.health.wa.gov.au/smokefree/docs/Mental_Health_Smoking_Checklist.pdf

Appendixes:

Appendixe-1: Some Variables definition, type, measurement method and scale

S/No	Variable	Definition	Qualitative		Quantitative		Variable		Measurement Method	Scale
			Ordinal	Nominal	Discrete	Continuous	Independent	Dependent		
1	Age	Year from birth to present				X	X		Questionnaire	Year
2	Gender	Being male and female		X			X		Questionnaire	Male/Female
3	Educational Status	It is the level of participant is attended the school or not attended at all	X				X		Questionnaire	Grade
4	Economical status	It is an individual's or family's economic and social position in relation to others, based on income and owning some properties	X				X		Questionnaire	Income/properties owning
5	Marital status	Whether a person is married or not and separated or divorced after marriage		X			X		Questionnaire	Marred/not or
6	Employment status	It is the part of an employment that is includes employed by government, NGOs or , private as temporary or permanently, and daily labor and		X			X		Questionnaire	Employment and status
7	TB knowledge	Patients knowledge cause TB diseases, availability of treatment, curability of the disease, duration of treatment, way of transmission, prevention, symptoms of the disease, not		X			X		Questionnaire	Correct/ incorrect/ don't know

S/No	**Variable**	**Definition**	**Qualitative**		**Quantitative**		**Variable**		**Measurement Method**	**Scale**
			**Ordinal**	**Nominal**	**Discrete**	**Continuous**	**Independent**	**Dependent**		
8	Health system factor	Is the factors that related with health care facilities (drug & laboratory supplies, supervision, continuous treatment and capacity building, quality of service, waiting time for treatment)		X			X		Questionnaire	Effective/ not
9	Health care worker	Is the relation between health care worker and patient (poor communication, lack of motivation, support, listening patients worry and patience)		X			X		Questionnaire	Good/ poor
10	Alcohol consumption	It is taking of alcoholic beverages like local beer, factory beer, wine, pasteurized draft etc.				X	X		Questionnaire	Likert
11	Tobacco smoking	It using of one or more of the following tobacco products (cigarettes, snuff, chewing tobacco, [illegible]		X			X		Questionnaire	Smoking/ not
12	Feeling better	It is diminish of TB signs and symptoms after treatment started and before full course treatment		X			X		Questionnaire	likert
13	Perceived susceptibility	TB patient's opinion of chances of getting both medication susceptible and resistant types of TB				X	X		Questionnaire	Likert
14	Perceived severity	TB patient's opinion of how serious both medication susceptible and resistant types of TB and their consequences are				X	X		Questionnaire	Likert
15	Perceived benefit	TB patients belief in the efficacy of the TB medication and on the importance of treatment adherence to reduce risk or seriousness of the				X	X		Questionnaire	Likert

S/No	Variable	Definition	Qualitative		Quantitative		Variable		Measurement Method	Scale
			Ordinal	Nominal	Discrete	Continuous	Independent	Dependent		
16	Perceived barrier	TB patient's opinion of the tangible and psychological costs of treatment adherence				X	X		Questionnaire	Likert
17	Cues to action	Factors that motivate or activate TB patient's readiness to be adherent on his/her treatment				X	X		Questionnaire	Likert
18	Self-efficacy	Confidence in TB patient's ability to follow his/her treatment correctly until complete				X	X		Questionnaire	Likert
19	Psychological distress	A combination of mental morbidity characterized by depression and anxiety				X	X		Questionnaire	Likert
20	Non-adherence	It is the patient's inability or refusal to take TB drugs as prescribed				X		X	Visual analog scale	Percentage (%)

6.2. Appendix 2. Questionnaires

Health Facility Name____________________ Date ______/_____/_____ **Confidential**

Title of the study: Psychosocial and Behavioral Determinants of TB Treatment Non-adherence; and Planning Educational and Psychosocial Intervention for Treatment Adherence in Ethiopia: Guided by Health Belief Model

Dear participant, my name is --------------------------, professionally I am medical doctor/ health officer/ nurse; and I am a worker of -- health center and I am collecting data for research project intended to identify ***psychosocial and behavioural determinants of TB treatment non-adherence and planning educational and psychosocial intervention to improve treatment adherence.*** Now, I want to ask you, your willingness to participate on this research by your full interest. The research is important for you and for general community to increase curability of TB disease and to prevent medication resistant types of TB germ by avoiding/decreasing factors that oblige TB patients to be non-adherent with their treatment. Also, I would like to tell you as you have full rights to participate or not participate on this study and as you have right to stop participation at any time on the process of study duration. Additionally, all information you will give us are confidential and used only for this study.

Dear participant, thank you for your cooperation in advance; and now I will read to you or you can read this written consent form; and you will sign for me as you agreed to participate in this study with your full will, after you understand the objective of the study, all procedures will be taken, and your benefits and rights.

6.2.1. Appendix 2.1. Sociodemographic Variables Questions

Site Name____ Date: ____/____/2014

Study Identification Number(SIDN):	TB Registration Book Number	
Dear interviewer, don't forget first to get participant's consent after you tell him/her about the objective and benefits of this study.		
Please ask the participant the following and tick ☐ for each question or write on the space provided		
1. Age:	-------------------Years	
2. Gender:	1= Male	☐
	2= Female	☐
3. Distance in average from home of participant to treatment center in Km:	---------------------Km	
4. Education level:	1 = Illiterate	☐
	2 = Informal Education	☐
	3 = 1-6 grade	☐
	4 = 7-8 grade	☐
	5 = 9-12 (9 – preparatory complete)	☐
	6 = Diploma graduate (2 or more years)	☐
	7 = Degree Graduate and above	☐
5. Marital status	1 = Unmarried living with mother &/or father	☐
	2 = Unmarried living lonely	☐
	3 = Combined (Living with boy/girl friend)	☐
	4 = Married living with Spouse	☐
	5 = Divorced<br>6 = Separated	☐
	4 = Any others (specify) -----------	☐

Study Identification Number(SIDN):	TB Registration Book Number (TBRBIDN):		
6. Properties of participant own or have the capacity to do without any difficulty.	1 = Owning house	1 = Yes	☐
		2 = No	☐
	2 = Owning refrigerator in home	1 = Yes	☐
		2 = No	☐
	3 = Owning Cupboard in home	1 = Yes	☐
		2 = No	☐
	4 = Owning satellite dish in home	1 = Yes	☐
		2 = No	☐
	5 = Ability to pay privet house rent	1 = Yes	☐
		2 = No	☐
	6 = Ability to pay government house rent	1 = Yes	☐
		2 = No	☐
	7 = Having electricity and water line in compound	1 = Yes	☐
		2 = No	☐
	8 = Ability to pay electricity and water bills per month	1 = Yes	☐
		2 = No	☐
	9 = Ability to eat food at least three times per a day	1 = Yes	☐
		2 = No	☐
	10 = Ability to pay social association like “Edir”	1 = Yes	☐
		2 = No	☐

Study Identification Number(SIDN):	TB Registration Book Identification Number (TBRBIDN):	
7. Employment condition	1 = Unemployed/jobless	☐
	2 = Employed by government permanently	☐
	3 = Employed by government temporarily	☐
	4 = Temporary employment in private company	☐
	5 = Permanent employment in private company	☐
	6 = Daily labor	☐
	7 = Self employed	☐
	8 = Employed but stopped because of TB disease	☐
8. Migration condition and plan in the future	1= Yes immigrated from rural	☐
	2 =No, living in original place	☐
9. If question number 9 is yes, ask the reason why he/she plan to change the current residency	1 = Personal interest	☐
	2 = Lack of money	☐
	3 = Lack of home	☐
	4 = Lack of transportation cost	☐
	5 = Lack of food	☐
	6 = Lack of support	☐

6.2.2. Appendix 2.2. Questions on TB Disease and Its Treatment Knowledge

Site Name____ Date ____/_____/2014

Study Identification Number(SIDN):	TB Registration Book Number (TBRBID):	
Please ask the participant the following and tick ☐ for each according to his/her answer		
1. TB disease is caused by germ/bacteria.	1 = Correct	☐
	2 = Incorrect	☐
	3 = Don't know	☐
2. TB disease can be caused by hard work, hereditary, common cold, cold water, alcohol consumption or tobacco smoking.	1 = correct	☐
	2 = Incorrect	☐
	3 = Don't know	☐
3. TB can transmit from infected person to uninfected person.	1 = Correct	☐
	2 = Incorrect	☐
	3 = Don't know	☐
4. TB is transmitted by infectious droplet spread by infected person, during coughing and sneezing.	1 = Correct	☐
	2 = Incorrect	☐
	3 = Don't know	☐
5. TB can transmit through, sharing eating utensil, kissing and shaking hand.	1 = Correct	☐
	2 = Incorrect	☐
	3 = Don't know	☐
6. TB can be prevented from transmission by, covering mouth and nose during coughing and sneezing, appropriate sputum dropping, opening windows in home, and in car while traveling	1 = Correct	☐
	2 = Incorrect	☐
	3 = Don't know	☐

Study Identification Number(SIDN):	TB Registration Book Number (TBRBIDN):	
7. The main TB symptoms are cough ≥ 2 weeks, chest pain, heavy night sweating, appetite loss, weight loss, extreme tiredness or fatigue and coughing up blood with sputum.	1 = Correct	☐
	2 = Incorrect	☐
	3 = Don't know	☐
8. There is effective medical treatment for TB disease.	1 = Correct	☐
	2 = Incorrect	☐
	3 = Don't know	☐
9. If treated properly, TB disease is curable.	1 = Correct	☐
	2 = Incorrect	☐
	3 = Don't know	☐
10. Total time of TB treatment is six month for new patient and eight for retreated patient.	1 = Correct	☐
	2 = Incorrect	☐
	3 = Don't know	☐
11. TB treatment is received infront of health care worker or somebody assigned to observe the treatment for 2 months.	1 = Correct	☐
	2 = Incorrect	☐
	3 = Don't know	☐
12. Currently TB treatment is received infront of health care worker or somebody assigned to observe the treatment for 6 months.	1 = Correct	☐
	2 = Incorrect	☐
	3 = Don't know	☐

Study Identification Number(SIDN):	TB Registration Book Number (TBRBIDN):	
13. TB medication is taken in home while coming per a month for drug refill and check up during continuation phase.	1 = Correct	☐
	2 = Incorrect	☐
	3 = Don’t know	☐
14. TB is curable with only six to eight weeks course of treatment.	1 = Correct	☐
	2 = Incorrect	☐
	3 = Don’t know	☐
15. Discontinuation of TB treatment is possible, if once pain related TB is stopped, even though the prescribed treatment duration has not been completed.	1 = Correct	☐
	2 = Incorrect	☐
	3 = Don’t know	☐
16. Holy water can cure TB disease correctly without medical treatment.	1 = Correct	☐
	2 = Incorrect	☐
	3 = Don’t know	☐
17. Traditional drugs are effective to cure TB disease without medical treatment.	1 = Correct	☐
	2 = Incorrect	☐
	3 = Don’t know	☐

Study Identification Number(SIDN):	TB Registration Book Number (TBRBN):	
18. Only eating good food can cure TB disease without medical treatment.	1 = Correct	☐
	2 = Incorrect	☐
	3 = Don't know	☐
19. TB treatment has follow up examinations during treatment period.	1 = Correct	☐
	2 = Incorrect	☐
	3 = Don't know	☐
20. TB treatment interruption (non-adherence or lost to follow up) is the main cause of medication resistant types of TB developed.	1 = Correct	☐
	2 = Incorrect	☐
	3 = Don't know	☐
21. TB treatment interruption is the main cause of treatment failure	1 = Correct	☐
	2 = Incorrect	☐
	3 = Don't know	☐
22. TB treatment interruption is the main cause of retreatment after completion of first line treatment.	1 = Correct	☐
	2 = Incorrect	☐
	3 = Don't know	☐
23. TB treatment interruption increases probability of disease transmission to healthy people.	1 = Correct	☐
	2 = Incorrect	☐
	3 = Don't know	☐
	TB Registration Book	

Study Identification Number(SIDN):	Number (TBRBIDN):	
24. TB treatment interruption increases probability of death because of TB	1 = Correct	☐
	2 = Incorrect	☐
	3 = Don't know	☐
25. Medication resistant TB types are types of TB which cannot cured by first line TB medications	1 = Correct	☐
	2 = Incorrect	☐
	3 = Don't know	☐
26. MDR- TB type has effective medical treatment.	1 = Correct	☐
	2 = Incorrect	☐
	3 = Don't know	☐
27. MDR- TB type is curable if treated properly.	1= Correct	☐
	2 = Incorrect	☐
	3 = Don't know	☐
28. Treatment period of MDR-TB is much longer than first line TB treatment.	1 = Correct	☐
	2 = Incorrect	☐
	3 = Don't know	☐
29. Treatment of MDR-TB type is given by admitting to treatment center for some period.	1 = Correct	☐
	2 = Incorrect	☐
	3 = Don't know	☐

Study Identification Number(SIDN):	TB Registration Book Number (TBRBN):	
30. TB medications have side effects.	1 = Correct	☐
	2 = Incorrect	☐
	3 = Don't know	☐
31. Gastritis, fatigability, nausea and blurred eye are some of TB medication side effect symptoms.	1 = Correct	☐
	2 = Incorrect	☐
	3 = Don't know	☐
32. TB treatment side effects have medical supportive treatment	1 = Correct	☐
	2 = Incorrect	☐
	3 = Don't know	☐
33. TB medication side effects usually subside gradually by themselves or by supportive treatment	1 = Correct	☐
	2 = Incorrect	☐
	3 = Don't know	☐
34. If TB patient has medication side effect, he/she has to be stop TB treatment.	1 = Correct	☐
	2 = Incorrect	☐
	3 = Don't know	☐
35. TB treatment adherence and not lost to follow up are very important to prevent medication resistant types of TB	1 = Correct	☐
	2 = Incorrect	☐
	3 = Don't know	☐

Study Identification Number(SIDN):	TB Registration Book Number (TBRBN):	
36. TB treatment adherence and not lost to follow up are very important to prevent treatment failure and drug resistant types of TB	1 = Correct	☐
	2 =Incorrect	☐
	3 = Don’t know	☐
37. TB treatment adherence and not lost to follow up are very important to prevent retreatment.	1 = Correct	☐
	2 = Incorrect	☐
	3 = Don’t know	☐
38. TB treatment non-adherence can lead to the development of Extensively Drug Resistant TB (XDR-TB) type of TB.	1 = Correct	☐
	2 = Incorrect	☐
	3 = Don’t know	☐

6.2.3. Appendix 2.3. Health Belief Model Domains Question

Study Site Name___________________________________ Date: _____/______/2014

Study Identification Number(SIDN):	TB Registration Book Number (TBRBID):
**Perceived Susceptibility**	
Please ask the participant and circle the selected level number, to what extent he/she agree or disagree with the following statement (where strongly disagree =1, disagree =2, Neutral =3, agree =4, strongly agree = 5)	
1. All human are at risk of acquiring medication resistant or susceptible TB type germ at any time.	1 2 3 4 5
2. I am at risk of acquiring medication resistant or susceptible TB type germ at any time	1 2 3 4 5
3. I am at higher risk of developing medication resistant types of TB than a person who is not on treatment	1 2 3 4 5
4. Children, old people and a person who has other diseases (HIV, Cancer, diabetics, etc.) are at higher risk of developing active type of TB than healthy adults.	1 2 3 4 5
5. If I do not complete my treatment correctly, I could develop medication resistant types of TB	1 2 3 4 5
6. If I am not take my treatment according to doctor prescription I could develop medication resistant types of TB	1 2 3 4 5
7. My family get higher chance to acquire medication sensitive or resistant types of TB germ from me, than general community	1 2 3 4 5
8. ***Ask only for Person Cero reactive for HIV:*** I get more chance to develop active TB, than any person without HIV	1 2 3 4 5
9. ***Ask only for Person Cero reactive for HIV:*** I get more chance to develop medication resistant types of TB, than person without HIV.	1 2 3 4 5

Study Identification Number(SIDN):	TB Registration Book Number (TBRBN):
**Perceived severity**	
Please ask the participant and circle the selected level number, to what extent he/she agree or disagree with the following statement (where strongly disagree =1, disagree =2, Neutral =3, agree =4, strongly agree = 5)	
1. If not treated properly, both medication susceptible and resistant types of TB cause serious health problems	1 2 3 4 5
2. Medication resistant types of TB may cause much serious health problem than medication susceptible type of TB	1 2 3 4 5
3. If I don't follow prevention methods correctly, TB is highly contagious disease which can infect my whole family members	1 2 3 4 5
4. Treatment of medication resistant types of TB have more serious side effects than first line TB treatment	1 2 3 4 5
5. Treatment period of medication resistant types of TB is much longer than medication susceptible type of TB	1 2 3 4 5
6. If we don't complete our treatment correctly, we may again get sick from TB disease	1 2 3 4 5
7. If TB disease is not treated properly, it has the capacity to kill human	1 2 3 4 5
8. TB is the most killer disease from infectious diseases which have effective treatment	1 2 3 4 5
9. Retreatment of TB disease increases drug side effects burden on the patient.	1 2 3 4 5

Study Identification Number(SIDN):	TB Registration Book Number (TBRBN):
**Perceived Barriers**	
Please ask the participant and circle the selected level of number, to what extent he/she agree or disagree with the following statement (where strongly disagree =1, disagree =2, Neutral =3, agree =4, strongly agree = 5)	
1. My interest obliged me not to take TB medication at all.	1 2 3 4 5
2. If pain (symptoms), due to TB disease disappear, stopping treatment is possible, even if the duration of treatment is not completed	1 2 3 4 5
3. TB medications have very serious side effects which cannot be treated.	1 2 3 4 5
4. TB medications side effects with HIV medications have serious side effects which may kill human.	1 2 3 4 5
5. Too many pills are dangerous to human and can damage abdominal organs.	1 2 3 4 5
6. TB drugs can weaken our body natural protection.	1 2 3 4 5
7. Coming every day for treatment is not necessary at all.	1 2 3 4 5
8. TB treatment appointment time is not convenient for TB patients.	1 2 3 4 5
9. Taking TB medication taking infront of health care worker/treatment supervisor is not necessary at all.	1 2 3 4 5
10. TB patients are not responsible to take their TB medication correctly.	1 2 3 4 5

Study Identification Number(SIDN):	TB Registration Book Number (TBRBN):
11. TB medication taking in empty stomach is very dangerous for health, so it is better to stop the medication when someone has no food to eat.	1 2 3 4 5
12. Eating quality food is mandatory to be cure from TB disease by TB medication.	1 2 3 4 5
13. I am hiding my disease condition, because, if the people may notice my disease status speak bad thing about me.	1 2 3 4 5
14. I don't like to be seen in TB clinic by somebody who knows me.	1 2 3 4 5
15. People connect TB disease with HIV; therefore I don't like to come here daily.	1 2 3 4 5
16. Support from family, friends and community is not very important to follow TB treatment correctly	1 2 3 4 5
17. Getting family, friends and community support is mandatory to complete my treatment correctly	1 2 3 4 5
18. Health care worker way of communication is very bad to come for treatment	1 2 3 4 5
19. Health care worker are unfriendly request most of the time a TB patient to stay away from them.	1 2 3 4 5
20. Registration fee for treatment is very expensive to pay for me and touches my economy seriously to complete my treatment correctly.	1 2 3 4 5
21. Lack of transportation cost is the one affecting me to complete my treatment correctly	1 2 3 4 5
22. Lack of food is very challenge for me to complete my treatment correctly	1 2 3 4 5

Study Identification Number(SIDN):	TB Registration Book Number (TBRBN):
23. Lack of permission from job place is very serious problem which may oblige me to interrupt my treatment	1 2 3 4 5
24. Lack of family is the big problem to complete my treatment correctly	1 2 3 4 5
25. My family insisted me to interrupt my treatment and advice me to go for holy water	1 2 3 4 5
26. Traditional medication is the one I like to take than TB drugs	1 2 3 4 5
27. My friends influenced me to interrupt my treatment sometimes to talk with them	1 2 3 4 5
28. Sometimes I interrupt my treatment to go for drink with my friends	1 2 3 4 5
29. Sometimes I interrupt TB treatment to smoke cigarette	1 2 3 4 5
30. My personal emotional conditions such as depression, motives, sadness, anger etc. obliged me to interrupt my treatment correctly	1 2 3 4 5
31. Forgetfulness obliged me to interrupt my treatment some times	1 2 3 4 5

**Perceived Benefits**	
Please ask the participant and circle the selected level number, to what extent he/she agree or disagree with the following statement (where strongly disagree =1, disagree =2, Neutral =3, agree =4, strongly agree = 5)	
1. If I take correctly and complete my treatment, TB medication could cure TB disease effectively.	1 2 3 4 5
2. TB treatment could support my natural body protection ability	1 2 3 4 5
3. TB treatment could enhance my quality of life.	1 2 3 4 5
4. If I take my medication properly, I will not develop medication resistant types of TB	1 2 3 4 5
5. If I get treated for TB correctly, TB could not spread to my family members	1 2 3 4 5
6. If I complete my treatment correctly, TB could not relapse to me, and I am free from retreatment	1 2 3 4 5
7. If I take my TB medication properly, my health will be recovered slowly.	1 2 3 4 5
8. If I take TB treatment properly, I will not die because of TB	1 2 3 4 5

Cues to action	
Please ask the participant and circle the selected number, to what extent he/she agree or disagree with the following statement (where strongly disagree =1, disagree =2, Neutral =3, agree =4, strongly agree = 5)	
1. My interest to cure motivated me to follow my treatment correctly.	1 2 3 4 5
2. The mass media affected my decision whether to complete my treatment correctly	1 2 3 4 5
3. Health care workers advice affected my decision whether to complete my treatment correctly.	1 2 3 4 5
4. My friends' advice and support affect my decision whether to complete my treatment correctly.	1 2 3 4 5
5. My family advice and support affect my decision whether to complete my treatment correctly.	1 2 3 4 5
6. My religion father advices motivate me to complete my treatment correctly.	1 2 3 4 5
7. Community supports motivate me to complete my treatment correctly.	1 2 3 4 5
8. Health care worker support and good communication motivate me to complete my treatment correctly.	1 2 3 4 5

Study Identification Number(SIDN):	TB Registration Book Number (TBRBN):
**Perceived Self-Efficacy**	
Please ask the participant and circle the selected number, to what extent he/she agree or disagree with the following statement (where strongly disagree =1, disagree =2, Neutral =3, agree =4, strongly agree = 5)	
1. I belief, I can take my medication correctly until I complete my follow up	1 2 3 4 5
2. I belief, I have capacity to tolerate TB medication side effects	1 2 3 4 5
3. I belief, I can request supportive treatment for TB drug side effects, before stopping my treatment	1 2 3 4 5
4. I belief, I can overcome people's bad speak about my disease, and I can come to treatment center	1 2 3 4 5
5. I belief, I can tolerate health care worker bad behavior, and I can take my treatment correctly	1 2 3 4 5
6. I belief, I have physical capability to come and take my treatment regularly	1 2 3 4 5
7. I belief, I can overcome peoples persuasions not to take my drug regularly	1 2 3 4 5
8. I belief, I can remove any kind of emotional condition which oblige me to interrupt/ stop my treatment	1 2 3 4 5
9. I belief, I can overcome cultural and religious thoughts (herbal medicine, holy water, eating quality food only enough) which oblige me to interrupt/stop my treatment	1 2 3 4 5
10. I belief, I can overcome my personal behaviors which motivate me to interrupt/stop my follow up (alcohol drinking, smoking, substances use,) until I finish my treatment correctly	1 2 3 4 5
11. I belief, I can complete my treatment correctly whether I get family, friends and community support or not.	1 2 3 4 5

Study Site Name______________________________ Date: _____/_____/2014

6.2.4. Appendix 2.4. Psychological Distress Question (Kessler Psychological Distress Scale (K-10))

Study Identification Number(SIDN):	TB Registration Book Number (TBBID):

Please ask the participant and tick the answer that is correct for he/she

Questions	All of the time (score 5)	Most of the time (score 4)	Some of the time (score 3)	A little of the time (score 2)	None of the time (score 1)
1. In the past 4 weeks, about how often did you feel tired out for no good reason?					
2. In the past 4 weeks, about how often did you feel nervous?					
3. In the past 4 weeks, about how often did you feel so nervous that nothing could calm you down?					
4. In the past 4 weeks, about how often did you feel hopeless?					
5. In the past 4 weeks, about how often did you feel restless or fidgety?					
6. In the past 4 weeks, about how often did you feel so restless you could not sit still?					
7. In the past 4 weeks, about how often did you feel depressed?					
8. In the past 4 weeks, about how often did you feel that everything was an effort?					
9. In the past 4 weeks, about how often did you feel so sad that nothing could cheer you up?					
10. In the past 4 weeks, about how often did you feel worthless?					

Study Site Name____ Date ___/_____/2014

6.2.5. Appendix 2.5. Alcohol Consumption History Questions (WHO Alcohol Use Disorder Identification Test (AUDIT))

<table>
<tr><td>Study Identification Number(SIDN):</td><td>TB Registration Book Number (TBBID):</td></tr>
<tr><td colspan="2">The Alcohol Use Disorders Identification Test: Interview Version

Read questions as written. Record answers carefully. Begin the AUDIT by saying “Now I am going to ask you some questions about your use of alcoholic beverages during this past year.” Explain what is meant by “alcoholic beverages” by using local examples of local beer, factory beer, Catical, kunduftu, wine, Pasteurized Draft/Jambo, etc. Code answers in terms of “standard drinks”. Place the correct answer number in the box at the right.</td></tr>
<tr><td>1. How often do you have a drink containing alcohol?

0 = Never [Skip to Qs 9-10]

1= Monthly or less

2 = 2 to 4 times a month

3 = 2 to 3 times a week

4 = 4 or more times a week

☐</td><td>2. How many drinks containing alcohol do you have on a typical day when you are drinking?

0 = 1 or 2

1 = 3 or 4

2 = 5 or 6

3 = 7, 8, or 9

4 = 10 or more

☐</td></tr>
<tr><td>3. How often do you have six or more drinks on one occasion?

0 = Never

1 = Less than monthly

2 = Monthly

3 = Weekly

4 = Daily or almost daily

Skip to Questions 9 and 10 if Total Score for Questions 2 and 3 = 0

☐</td><td>4. How often during the last year have you found that you were not able to stop drinking once you had started?

0 = Never

1 = Less than monthly

2 = Monthly

3 = Weekly

4 = Daily or almost daily

☐</td></tr>
</table>

Study Identification Number(SIDN):	TB Registration Book Number (TBRBN):
5. How often during the last year have you failed to do what was normally expected from you because of drinking?<br><br>0 = Never<br><br>1= Less than monthly<br><br>2 = Monthly<br><br>3 = Weekly<br><br>4 = Daily or almost daily<br><br>☐	6. How often during the last year have you needed a first drink in the morning to get yourself going after a heavy drinking session?<br><br>0 = Never<br><br>1 = Less than monthly<br><br>2 = Monthly<br><br>3 = Weekly<br><br>4 = Daily or almost daily<br><br>☐
7. How often during the last year have you had a feeling of guilt or remorse after drinking?<br><br>0 = Never<br><br>1 = Less than monthly<br><br>2 = Monthly<br><br>3 = Weekly<br><br>4 = Daily or almost daily<br><br>☐	8. How often during the last year have you been unable to remember what happened the night before because you had been drinking?<br><br>0 = Never<br><br>1 = Less than monthly<br><br>2 = Monthly<br><br>3 = Weekly<br><br>4 = Daily or almost daily<br><br>☐
9. Have you or someone else been injured as a result of your drinking?<br><br>0 = No<br><br>2 = Yes, but not in the last year<br><br>4 = Yes, during the last year<br><br>☐	10. Has a relative or friend or a doctor or another health worker been concerned about your drinking or suggested you cut down?<br><br>0 = No<br><br>2 = Yes, but not in the last year<br><br>4= Yes, during the last year<br><br>☐
Sum up specific items values to calculate total, then record here ☐	
NB: If total is greater than recommended cut-off, consult User's Manual (*Recommended cut-off scores for person less than 65 is 8 and for person above 65 is 7*)	

Study Site Name_________________________________ Date: ____/_____/2014

6.2.6. Appendix 2.6. Tobacco Smoking and Nicotine Dependency Questions

Study Identification Number(SIDN):	TBBN:	
Please ask participant, the following question and tick ☐ for each question		
1. Do you currently use one or more of the following tobacco products (cigarettes, snuff, chewing tobacco, cigars, hookah etc.)?	1 = Ye:	☐
	2 = No:	☐
2. In the past month, how often have you used one or more of the following tobacco products (cigarettes, snuff, chewing tobacco, cigars, hookah etc.)?	1 = Once or twice	☐
	2 = Weekly	☐
	3 = Almost daily	☐
	4 = Daily	☐
3. How soon after awaking do you smoke your first cigarette?	0 = 60+ minutes	☐
	1 = 31-60 minutes	☐
	2 = 5-30 minutes	☐
	3 = Within 5 minutes	☐
4. How many cigarettes a day do you smoke?	0 = 10 or less	☐
	1 = 11 – 20	☐
	2 = 21 – 30	☐
	3 = 31 or more	☐
Total score will be calculated only for question 3 and 4 to estimate nicotine dependency<br>**Total Score =**		

6.2.7. Appendix 2.7. TB Treatment History and TB type Questions

Please register type and treatment history of TB from TB registration book and tick ☐ box		
1. Types of TB	1 = PTB^{+}	☐
	2 = PTB^{-}	☐
	3 = EPTB	☐
	4 = MDR-TB	☐
2. TB treatment history	1 = New	☐
	2 = Retreatment after failure	☐
	3 = Retreatment after default	☐
	4 = Retreatment after completion	☐
	5 = Retreatment after relapse	☐

6.2.8. Appendix 2.8. HIV Sero-status and ART Status Questions

Study Identification Number(SIDN):	TB Registration Book Identification Number (TBRBN):	
Please ask the participant his/her HIV Cero status and ART condition then tick ☐ box		
1. HIV Cero status	1 = Reactive	☐
	2 = None reactive	☐
2. ART status	1 = Currently on ART	☐
	2 = On ART before starting TB treatment , but currently stopped	☐
	3 = Not start at all	☐

6.2.9. Appendix 2.9. Treatment Adherence level Questions

Study Site Name________________________________ Date: _____/______/2014

Study Identification Number(SIDN):	TB Registration Book Identification Number (TBRBN):

1. Please ask the participant to guess and put capital “X” in the box below based on how much he/she has been taken his/her anti-TB medication correctly (without missing) in the past 30 days. Also, tell the participant 0% means he/she has not taken his/her anti-TB medication, 50% means he/she has taken half of his/her anti-TB medication and 100% means he/she has taken every single dose of his/her anti-TB medication.

0% 10% 20%30%40%50% 60%70% 80% 90% 100%

Estimate percent indicated ☐☐

2.

Please see the participant’s follow up card or TB registration book , to find how may day/s the participant was not taken the medication/ miss an appointment in last 30 days, then register the sum count in the box	Sum of days not taken/ missed = -------------------

NB: Dear data collectors don’t forget to sign on each of filled questionnaire after rechecked as all questions are answered.

Data collector, Name and signature: ---Date: ------/----/2014

Tell Phone number of data collector: +2519---

Data Quality Checked by: Name and signature: ---------------------------------, Date: -----/----/2014

7. Appendix 3. Patient Information and Consent Sheets

7.1. Appendix 3.1. Patient Information Sheet

Dear participant: We would like to ask you to take part in this study which aimed to assess the ***psychosocial and behavioural determinates of TB treatment non-adherence and to plan educational psychosocial intervention for treatment adherence*** which helps to prevent treatment failure and medication resistant types of TB. This study protocol and consent form has been approved by the Tehran University of Medical Sciences Research Ethics Review Board TUMS RERB), Teheran, Iran; and Ethiopian Health and Nutrition Research Institute Research and Ethical Clearance Committee (RECC) to make sure that your rights and autonomy are protected.

If you agree to participate in this study, these are what will be happen:

- You tell interviewer that you agree to be part of the project.
- You will be answer questions about your socio-demographic, TB and its treatment for 30:00 minute
- You may be provided education program that takes 30:00 minutes for two month per a week
- No identifying information about you, will be collected for this project, except some socio-demographic and TB disease and its treatment related information, such as your age, gender, education level, economic level, alcohol consumption and tobacco smoking history, TB disease and its treatment knowledge, some mental health status, treatment adherence level and health care worker level of communication with you and service quality of this health centre now, at the beginning of the study and at the end of your treatment
- This study result will be reported through publication with your full approval. During, publications of its result, no any identifying information about you or any other participant will appear in the publishing report.
- Data collected for this study will not be used for other purpose, without your consent.

- Regarding risk or discomfort: there is no any risk or discomfort in this study procedure.
- Regarding benefits of the study, you as individual may get comprehensive knowledge about TB disease, its treatment and the consequences of treatment non-adherence. As general benefit, your participation in this study will greatly benefit for the improvement of TB control program at national and international level based on this study result report.

Project Manager: Habteyes Hailu Tola

Phone Number: -----------------------------------

Work Address: Ethiopian Health and Nutrition Research Institute, Addis Ababa, Gulele sub-city, Arbegnoch Street.

7.2. Appendix 3.2. Written Informed Consent Sheet

Dear participant, if you are agreed to take part in this study based on the information given to you, please listen or read this form one by one and tick every box to show your agreement on each points and sign the consent sheets at the end of this form. If there is any unclear point, don't hesitate to ask question until you understand it to make your decision by your own interest.

1. I know the objective and procedure of this study after I have read, or it was read to me, the information sheet concerning this study and I understand what will be required of me if I take part in the study.	☐
2. I understand that being participation on this study is voluntary; confidentiality of my personal information is guaranteed; and as I have the right to get full treatment/care without participating on this study for my TB disease.	☐
3. I understand that at any time I have the right to withdraw from this study without giving a reason and without affecting my normal treatment/care.	☐
4. I know as data collector, collects information regarding TB disease and its treatment from me after he/she explain for me the procedure of collection, and as it takes 30 minutes to complete the whole procedure.	☐
5. I know the potential benefit of this study for national and international TB control program through enhancing treatment adherence, that is important to prevent medication resistant types of TB and treatment failure	☐
6. The interviewer explain for me as there is no any risk or discomfort, and extra treatment rather than normal TB treatment which I am taking	☐
7. I understand that as information collected from me are confidential, and they will be reported with my approval and the information will be reported are only the result without my personal information.	☐
8. I know there is no extravagant to me without time taken for interview, counselling and health education.	☐
9. Project manager promised me, as I can communicate him, if there is any problem and help regarding TB treatment to guide me for appropriate solution	☐

10. I know that, if there are any physical, mental problems due to participating in the study, all responsibilities are come to project manager.	☐
11. I know that, if there is any disagreement with the procedure of the study I will appear to research ethical clearance board of Tehran University of Medical Sciences – International Campus, Qods Street, School of Public Health, Tehran, Iran or to Ethiopian Public Health Institute, Gulele Sub-city, Arbegnoch Street. Addis Ababa, Ethiopia with written or verbal appeal.	☐
❖ This form and informed consent will be filled and signed in two copies, and then one copy will be provided for participant and the other for project manager.	

I understand all the information given above and I agreed to participate in this study by my full interest. And I assure my agreement by my official signature.

Signature: ---, Date: -----------/--------/2014

Participant Phone Address if possible: -----------------------------------

I project manager Habteyes Hailu Tola, agreed on all commitment on this form to fulfill all safety procedure, right and benefit for the participants, and then I assure my agreement by my official signature

Project Manager Name: Habteyes H. Tola

Phone Number: ----------------------------------

In country work address: Ethiopian Public Health Institute, Ethiopia, Addis Ababa, Gulele sub-city, Arbegnoch Street.

Signature: --, Date: -------/------/2014

8. Appendix 4. Data management and Study Procedure Flow Diagram

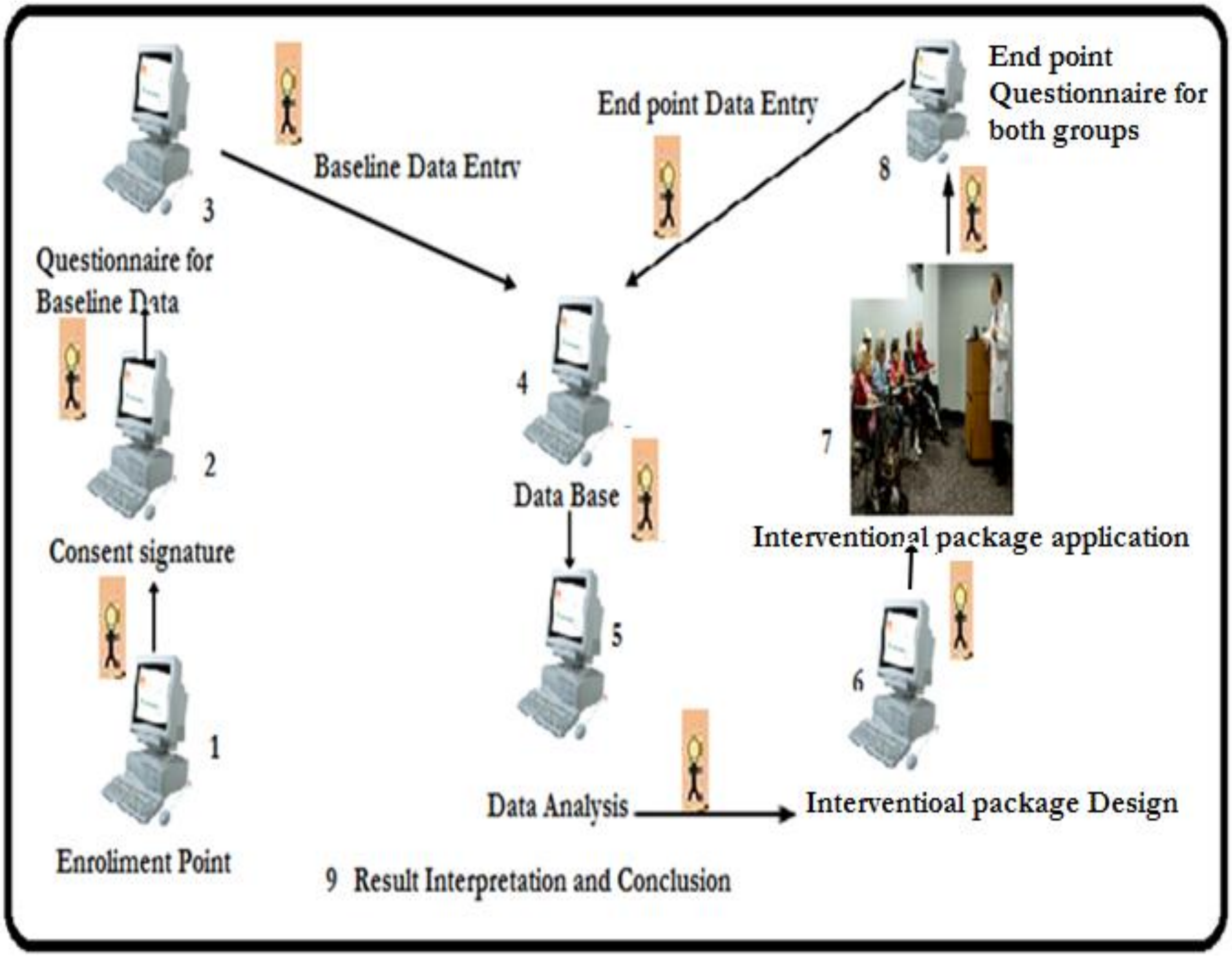

Supplement: S1 Protocol — (PDF) [file pone.0155147.s002.pdf]
